# Supplementary material for: DEveloping Tests for Endometrial Cancer deTection (DETECT): protocol for a diagnostic accuracy study of urine and vaginal samples for the detection of endometrial cancer by cytology in women with postmenopausal bleeding
Source: BMJ Open. 2021 Jul 28;11(7):e050755. doi: 10.1136/bmjopen-2021-050755 (PMC8320249; doi:10.1136/bmjopen-2021-050755)
Supplement: Supplementary data [file bmjopen-2021-050755supp001.pdf]

Appendix 1. Acceptability questionnaire

How would you describe your education level?

Primary School (Up to age 11) ☐

Secondary school (Up to age 16) ☐

Higher Education or above (A Levels/ University) ☐

	Yes	No
**Have you used any vaginal creams/ pessaries before?**	☐	☐
**Have you ever used tampons?**	☐	☐
**Have you given a urine sample before?**	☐	☐
**Have you ever used a home pregnancy test?**	☐	☐

Have you been sexually active?

Within the last month ☐

Within the last year ☐

More than a year/ not sexually active ☐

Would rather not say ☐

How would you describe the amount of discomfort experienced during the vaginal washing?

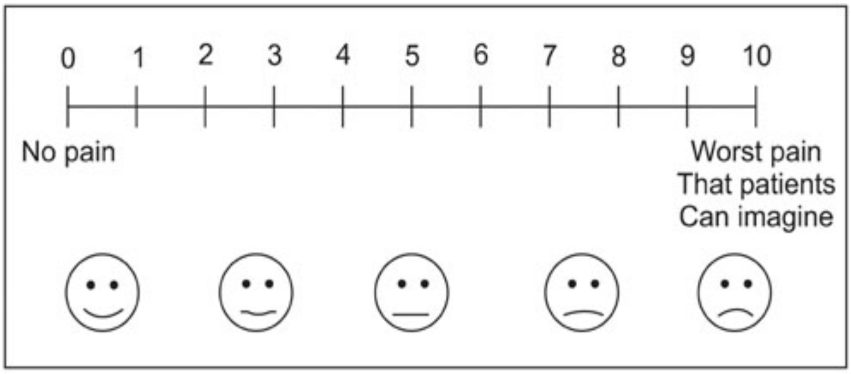

How would you describe the amount of discomfort experienced during pelvic examination?

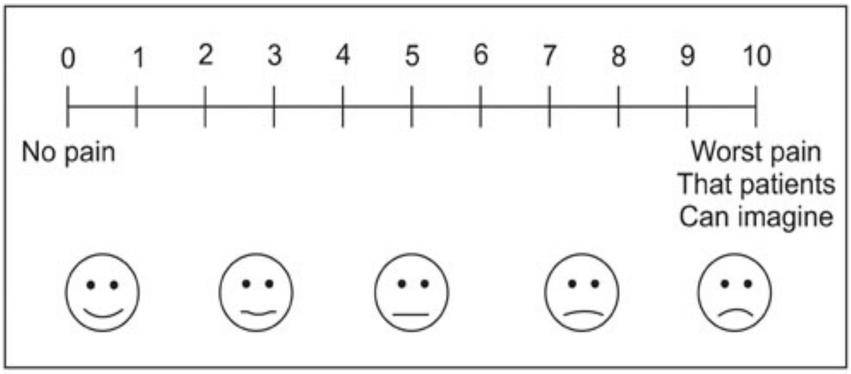

How would you describe the amount of discomfort experienced during ultrasound scan?

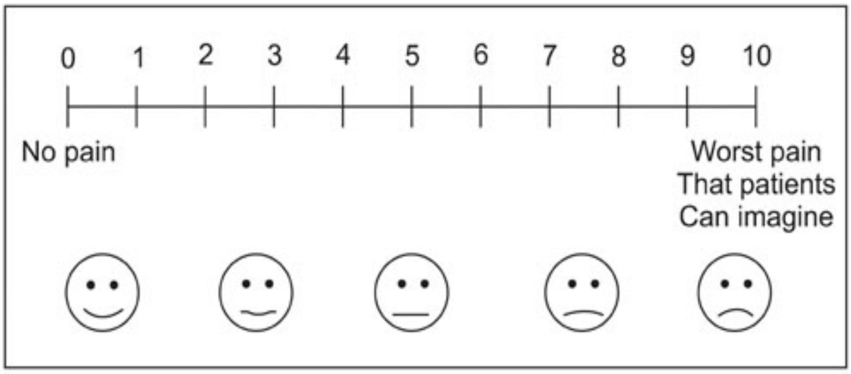

How would you describe the amount of discomfort experienced during hysteroscopy?

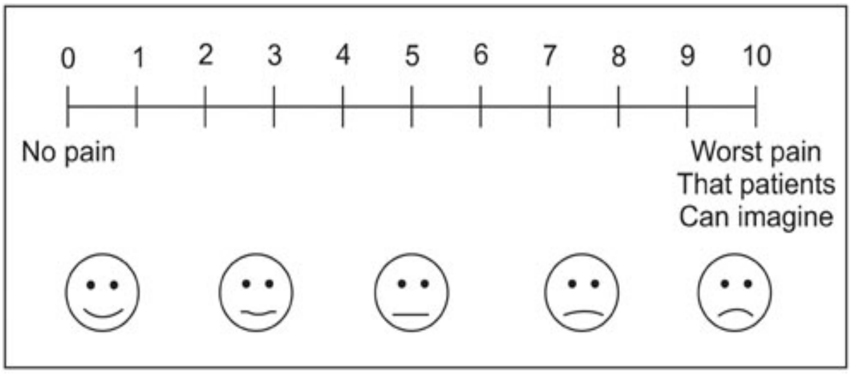

Jones ER, *et al. BMJ Open* 2021; 11:e050755. doi: 10.1136/bmjopen-2021-050755

How would you describe the amount of discomfort experienced during endometrial biopsy sampling?

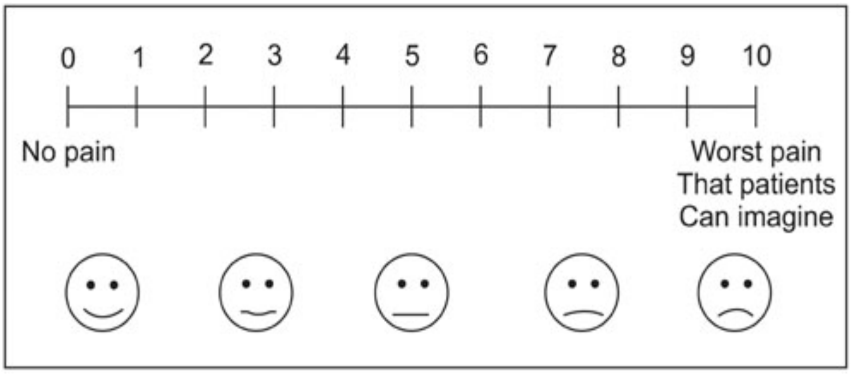

If needed how likely are you to have the following investigations again?

	**Definitely not**	**Probably not**	**Not sure**	**Probably**	**Definitely**
Ultrasound scan	☐	☐	☐	☐	☐
Vaginal washing	☐	☐	☐	☐	☐
Urine sample	☐	☐	☐	☐	☐
Pelvic examination	☐	☐	☐	☐	☐
Hysteroscopy	☐	☐	☐	☐	☐
Endometrial biopsy	☐	☐	☐	☐	☐

Jones ER, *et al. BMJ Open* 2021; 11:e050755. doi: 10.1136/bmjopen-2021-050755
