# Supplementary material for: DEveloping Tests for Endometrial Cancer deTection (DETECT): protocol for a diagnostic accuracy study of urine and vaginal samples for the detection of endometrial cancer by cytology in women with postmenopausal bleeding
Source: BMJ Open. 2021 Jul 28;11(7):e050755. doi: 10.1136/bmjopen-2021-050755 (PMC8320249; doi:10.1136/bmjopen-2021-050755)
Supplement: Author's manuscript [file bmjopen-2021-050755.draft_revisions.pdf]

BMJ Open

BMJ Open is committed to open peer review. As part of this commitment we make the peer review history of every article we publish publicly available.

When an article is published we post the peer reviewers' comments and the authors' responses online. We also post the versions of the paper that were used during peer review. These are the versions that the peer review comments apply to.

The versions of the paper that follow are the versions that were submitted during the peer review process. They are not the versions of record or the final published versions. They should not be cited or distributed as the published version of this manuscript.

BMJ Open is an open access journal and the full, final, typeset and author-corrected version of record of the manuscript is available on our site with no access controls, subscription charges or pay-per-view fees (http://bmjopen.bmj.com).

If you have any questions on BMJ Open's open peer review process please email info.bmjopen@bmj.com

BMJ Open

DEveloping Tests for Endometrial Cancer deTection (DETECT): Protocol for a diagnostic accuracy study of urine and vaginal samples for the detection of endometrial cancer by cytology in women with postmenopausal bleeding

Journal:	*BMJ Open*
Manuscript ID	bmjopen-2021-050755
Article Type:	Protocol
Date Submitted by the Author:	27-Feb-2021

Complete List of Authors:	Jones, Eleanor ; The University of Manchester, Division of Cancer Sciences<br>Carter, Suzanne; The University of Manchester<br>O'Flynn, Helena; The University of Manchester<br>Njoku, Kelechi; The University of Manchester<br>Barr, Chloe ; The University of Manchester, Department of Obstetrics and Gynaecology<br>Narine, Nadira; Manchester University NHS Foundation Trust<br>Shelton, David; Manchester University NHS Foundation Trust<br>Rana, Durgesh; Manchester University NHS Foundation Trust<br>Crosbie, Emma; The University of Manchester

Keywords:	Gynaecological oncology < GYNAECOLOGY, CYTOPATHOLOGY, PRIMARY CARE

SCHOLARONE™
Manuscripts

I, the Submitting Author has the right to grant and does grant on behalf of all authors of the Work (as defined in the below author licence), an exclusive licence and/or a non-exclusive licence for contributions from authors who are: i) UK Crown employees; ii) where BMJ has agreed a CC-BY licence shall apply, and/or iii) in accordance with the terms applicable for US Federal Government officers or employees acting as part of their official duties; on a worldwide, perpetual, irrevocable, royalty-free basis to BMJ Publishing Group Ltd ("BMJ") its licensees and where the relevant Journal is co-owned by BMJ to the co-owners of the Journal, to publish the Work in this journal and any other BMJ products and to exploit all rights, as set out in our licence.

The Submitting Author accepts and understands that any supply made under these terms is made by BMJ to the Submitting Author unless you are acting as an employee on behalf of your employer or a postgraduate student of an affiliated institution which is paying any applicable article publishing charge ("APC") for Open Access articles. Where the Submitting Author wishes to make the Work available on an Open Access basis (and intends to pay the relevant APC), the terms of reuse of such Open Access shall be governed by a Creative Commons licence – details of these licences and which Creative Commons licence will apply to this Work are set out in our licence referred to above.

Other than as permitted in any relevant BMJ Author's Self Archiving Policies, I confirm this Work has not been accepted for publication elsewhere, is not being considered for publication elsewhere and does not duplicate material already published. I confirm all authors consent to publication of this Work and authorise the granting of this licence.

For peer review only

DEveloping Tests for Endometrial Cancer deTection (DETECT): Protocol for a diagnostic accuracy study of urine and vaginal samples for the detection of endometrial cancer by cytology in women with postmenopausal bleeding

Eleanor R. Jones[1], Suzanne Carter[1], Helena O'Flynn[1], Kelechi Njoku[1], Chloe E. Barr[1,2], Nadira Narine[3], David Shelton[3], Durgesh Rana[3], Emma J. Crosbie[1,2]*

[1]Gynaecological Oncology Research Group, Division of Cancer Sciences, Faculty of Biology, Medicine and Health, University of Manchester, Manchester, UK

[2]Department of Obstetrics and Gynaecology, St Mary's Hospital, Manchester University NHS Foundation Trust, Manchester Academic Health Science Centre, Manchester, UK

[3]Manchester Cytology Centre, Manchester University Hospitals NHS Foundation Trust, Manchester Academic Health Science Centre, Manchester, UK

*Corresponding author: Professor Emma Crosbie, Division of Cancer Sciences, University of Manchester, School of Medical Sciences, Faculty of Biology, Medicine and Health, 5th Floor Research, St Mary's Hospital, Oxford Road, Manchester M13 9WL. Email: emma.crosbie@manchester.ac.uk. Phone. 0161 701 6942

International Standard Randomised Controlled Trial Number: ISRCTN58863784

Key words: endometrial cancer, postmenopausal bleeding, early detection, non-invasive sampling, diagnostic tool, cytology

For peer review only

Abstract

Introduction: Postmenopausal bleeding (PMB), the red flag symptom for endometrial cancer, triggers urgent investigation by transvaginal ultrasound scan, hysteroscopy and/or endometrial biopsy. These investigations are costly, invasive and often painful or distressing for women. In a pilot study, we found that voided urine and non-invasive vaginal samples from women with endometrial cancer contain malignant cells that can be identified by cytology. The aim of the DETECT study is to determine the diagnostic test accuracy of urine and vaginal cytology for endometrial cancer detection in women with PMB.

Methods and analysis: This is a multi-centre diagnostic accuracy study of women referred to secondary care with PMB. Eligible women will be asked to provide a self-collected voided urine sample and a vaginal sample collected with a Delphi screener before routine clinical procedures. Pairs of specialist cytologists, blinded to participant cancer status, will assess and classify samples independently, with differences settled by consensus review or involving a third cytologist. Results will be compared with clinical outcomes from standard diagnostic tests. A sample size of 2,000 women will have 80% power to establish a sensitivity of vaginal samples for endometrial cancer detection by cytology of ≥85% ±7%, assuming 5% endometrial cancer prevalence. The primary objective is to determine the diagnostic accuracy of vaginal samples for endometrial cancer detection by cytology. Secondary objectives include the diagnostic accuracy of urine and combined urogenital cytology, and the acceptability of urine and vaginal sampling to women.

Ethics and dissemination: This study has been approved by the North West-Greater Manchester West Research Ethics Committee (16/NW/0660) and the Health Research Authority. Results will be disseminated through publication in peer-reviewed scientific journals, presentation at conferences and via charity websites.

For peer review only

For peer review only - http://bmjopen.bmj.com/site/about/guidelines.xhtml

Strengths and limitations of this study

- This is a prospective evaluation of a novel, non-invasive endometrial cancer detection tool that could transform diagnostic pathways for women with PMB
- Samples will be taken prior to routine clinical procedures to avoid inadvertent contamination of samples by iatrogenically-dislodged endometrial cells
- Cytologists are blinded to participant cancer status until they provide their consensus report
- Follow up of participants will ensure missed cancer diagnoses are minimised
- Recruitment is limited to women with PMB and results may not be applicable to premenopausal women or those with atypical presentations of endometrial cancer

For peer review only

INTRODUCTION

In the UK, over 9,000 women are diagnosed with endometrial cancer every year (1). The red flag symptom for endometrial cancer is postmenopausal bleeding (PMB) (2). A woman with PMB is referred by her GP on the urgent 'suspected cancer' pathway to a rapid access gynaecology clinic, where she is offered a series of invasive, unpleasant and often painful tests to rule out endometrial cancer (3). These include a transvaginal ultrasound scan, an outpatient hysteroscopy and an endometrial biopsy (4). Together, these tests cost the NHS around £750/woman (5). PMB is extremely common and only 5-10% of women with PMB are ultimately diagnosed with endometrial cancer (6, 7). Indeed, it has been estimated that 5% of all GP referrals to gynaecology, as many as 150,000 women per year in the UK alone, relate to PMB (5). A simple, non-invasive test deployed in primary care to target those at risk of endometrial cancer for invasive testing, whilst safely reassuring the vast majority of healthy women, could transform diagnostic pathways for endometrial cancer. In the UK, it would save thousands of women every year from the psychological and physical sequelae of invasive tests and create substantial cost savings for the NHS (potential saving in excess of £100M/year).

The development of novel non-invasive detection tools was voted the most important research priority for detecting cancer early in the recently completed James Lind Alliance Priority Setting Partnership (8). In endometrial cancer, the anatomical continuity between the uterus and the lower genital tract facilitates the collection of shed tumour cells using non-invasive sampling methodologies (9-13). We found that shed tumour cells can be collected from the vagina by gentle lavage using the Delphi screener, and from voided urine samples, which are inevitably contaminated by endometrial debris in women with uterine bleeding (14). These cells can be distinguished from normal squamous, urothelial and glandular cells of the urogenital tract by cytology, although certain benign mimics (e.g. polyps, atrophy) can cause difficulties with interpretation. In our pilot study of 113 women with unexplained PMB, urine and/or vaginal cytology showed 100% sensitivity and 100% negative predictive value for endometrial cancer detection; it identified all 7 cancers (4 endometrial, 1 cervical, 1 ovarian and 1 bladder) for a 11% false positive rate. Furthermore, mean pain scores were significantly lower for vaginal sampling (1.61, SD 2.04) than for diagnostic hysteroscopy (4.28, SD 2.61, p<0.001) and endometrial biopsy (4.88, SD 3.49, p<0.001), respectively (14). Thus urogenital cytology has considerable potential as a well tolerated 'rule out' test to enable quick reassurance for most women who present to primary care with PMB, and urgent referral for those who test positive. To confirm its clinical utility for endometrial cancer detection, urine and vaginal cytology must now be tested in a large prospective study of women undergoing investigation for PMB. The aim of the DEveloping Tests for Endometrial Cancer deTection (DETECT) study is to estimate the diagnostic

For peer review only - http://bmjopen.bmj.com/site/about/guidelines.xhtml

accuracy of urine and vaginal samples for endometrial cancer detection by cytology in women with PMB.

METHODS

This protocol is reported in accordance with Standards for Reporting of Diagnostic Accuracy Studies (STARD) guidelines (15).

Study design

DETECT is a prospective multicentre diagnostic accuracy study of urine and vaginal cytology for endometrial cancer detection in women with PMB (Study schema, Figure 1).

Participants and recruitment

Consecutive women will be recruited from gynaecology clinics at seven hospital sites across the North West of England: St Mary's Hospital, Trafford General Hospital, Wythenshawe Hospital, Royal Oldham Hospital, North Manchester General Hospital, Fairfield General Hospital and Tameside Hospital.

Inclusion criteria

1. Women who have been referred to secondary care for investigation of PMB
2. Written, informed consent to participate

Exclusion criteria

1. Abnormal bleeding before the menopause
2. Previous diagnosis of endometrial cancer
3. Previous hysterectomy
4. Mirena coil in situ or removed within the last 3 months
5. Any other condition that would compromise participant safety or data integrity

PMB will be defined as vaginal bleeding that occurs more than 12 months after menstruation has stopped due to menopause.

Participant withdrawal

Participants may withdraw from the study at their own request or at the discretion of the Investigator. Withdrawal from the study will not affect patient care.

For peer review only

For peer review only - http://bmjopen.bmj.com/site/about/guidelines.xhtml

Sample size

With a sample size of ~2,000 women, and an endometrial cancer prevalence of 5% (5), there will be approximately 1,900 women without endometrial cancer and 100 women with endometrial cancer. The study will have 80% power to determine the sensitivity of the test at ≥85% +/-7% and the specificity of the test at ≥85% +/-2%. At 85% specificity, around 1,630 women will have a negative test result, giving the test a negative predictive value (NPV) of 99.1% (98.5%, 99.4%). These estimates originate from the pilot study, where sensitivity and specificity were both >85%. The prevalence of endometrial cancer will determine the final sample size. If the prevalence of endometrial cancer is greater than 5%, fewer than 2,000 women will be needed; if it is less than 5%, more women will be needed to determine the sensitivity of the test at ≥ 85% +/-7%.

Study duration

In the pilot study, 90% of eligible women agreed to participate. Women will be recruited between September 2018 and September 2021 with clinical outcome data collected until March 2022. To achieve the recruitment target of ~2,000 women, approximately 20 women will be recruited per week across the seven hospital sites. A temporary pause on recruitment to the study was initiated in March 2020 because of the COVID-19 pandemic, with restrictions still in place at the time of this submission.

Invitational procedure

Eligible women will be identified from referral letters and clinic lists by hospital clinical staff. A letter inviting participation and a detailed participant information sheet (PIS) will be sent by post to their home address. Potential participants will have the opportunity to discuss the study over the telephone with members of the study research team prior to their appointment. Due to the urgent nature of referrals, some women are offered short notice clinical appointments by telephone. Where there is insufficient time for invitational material to be received by post, women will be informed about the study via telephone and/or invited to participate on arrival at the clinic. Women will be invited to read the PIS and ask questions about the study before providing written, informed consent to participate.

Baseline clinical data

The following demographic and baseline clinical data will be obtained: age, ethnicity, socioeconomic status, education level, body mass index (BMI), smoking status, parity, age of menopause, use of contraceptives, hormone replacement therapy (HRT) or tamoxifen, history of hypertension,

For peer review only

hypercholesterolaemia, diabetes mellitus, polycystic ovary syndrome, thyroid disease, coagulopathy, Lynch syndrome, cervical screening history, endometrial hyperplasia, and personal or family history of cancer. Women will be asked in detail about their help seeking behaviour and the onset, duration and extent of their PMB.

Index tests

The timing of urine and vaginal sampling is important to ensure validity of the results. Both research samples will be taken before any routine clinical procedures to avoid inadvertent contamination with iatrogenically-dislodged endometrial cells (Study schema, Figure 1). Of the two research samples, urine will be collected first because vaginal sampling will remove naturally shed uterine debris from the lower genital tract and affect interpretation of the results.

Urine samples

Using both written and verbal instructions, we will ask women to bring to clinic a first catch sample of their first urinary void of the day, collected in a sterile pot. A second voided urine sample will be collected by the participant upon arrival at the clinic, before any other research or clinical procedures are carried out.

Vaginal sample

The vaginal sample will be taken by the research practitioner using a Delphi screener (Rovers Medical Devices, OSS, Netherlands) (16) according to the following protocol, with the participant in the supine position, knees bent and legs apart. The Delphi screener is inserted into the posterior fornix of the vagina and 3ml saline expelled from its reservoir by depressing the plunger for three seconds. The sample is then collected by suction following release of the plunger whilst slowly rotating and withdrawing the device. A dry pot at the introitus collects any residual fluid. Additional samples are obtained by re-filling the reservoir with saline and repeating the steps above until clear fluid is obtained (maximum of three times).

Sample handling

Urine samples will be tested for haematuria by dipstick. Urine and vaginal samples will be fixed with equal volumes of BD CytoRich™ Red Preservative (Becton, Dickinson and Company, USA) to preserve cellular integrity, prevent degradation and inhibit bacterial overgrowth. Samples will be sent to the Manchester Cytology Centre at the Manchester University NHS Foundation Trust (MFT). Samples will be anonymised and labelled with sample type (urine or vaginal fluid) and a

For peer review only - http://bmjopen.bmj.com/site/about/guidelines.xhtml

unique study identifier (study ID) to prevent accidental unblinding of the cytopathology team. With the exception of whether the participant is taking exogenous hormones, all clinical data will be withheld from the cytology team until consensus results are received. Second urine samples will be fixed with BD CytoRich™ Red and stored at 4°C until the first urine sample has undergone cytological assessment. This second sample will undergo cytological review if the first urine sample is inadequate for analysis or post hoc, if the first urine sample is false negative for endometrial cancer detection. Residual samples will be stored in the MFT Biobank for future biomarker discovery work. Samples will either be embedded in agar cell blocks to preserve cellular integrity for future immunohistochemistry or centrifuged to pellet the cellular material. The resulting pellet plus aliquots of the supernatant will be stored at -80°C.

Cytological assessment

Samples will be centrifuged at 3000RPM for 5 minutes, supernatant decanted and the pellet resuspended in 6mls CytoRich™ Red. After 1 hour, the fixed sample will be centrifuged at 1500RPM for 10 minutes, supernatant decanted and the remaining pellet prepared into a liquid based cytology Papanicolaou stained slide using the BD prepstain (Becton Dickinson UK Limited) according to the manufacturer's instructions. The stained slide will be dehydrated in two changes of industrial methylated spirits, cleared in two changes of xylene and cover-slipped. Two observers, a consultant cytopathologist and a consultant Biomedical Scientist (BMS), will review each slide independently and record their results. A second consultant cytopathologist will review any discrepant cases, which will be settled by consensus review at a multi-headed microscope. Final cytology results will be logged under the unique study ID on the study database. Intra- and inter-observer variability will be determined by blinded, independent review of a random selection of anonymised test positive, test negative, complex and discrepant cases at the end of the study.

Classification of cytology results

Cytology slides will be reported according to the classification system shown in Table 1. For the primary analysis, suspicious, adenocarcinoma or malignant (other) cytology results will be considered positive. Glandular, atypical cells of uncertain significance (ACUS) and no malignant cells seen (NMCS) will be considered negative. Unsatisfactory results will not be classified as either positive or negative and the participant will be invited to provide a second sample for cytological analysis. Secondary analysis will include cancers of other urogenital sites (cervix, vagina, ovary, fallopian tube, bladder or kidney). Sensitivity analysis will consider the diagnostic

For peer review only - http://bmjopen.bmj.com/site/about/guidelines.xhtml

performance of positive urogenital cytology that includes glandular and ACUS results as potentially malignant findings.

Table 1: Cytological classification

Cytology result	Cytological findings	Primary analysis	Sensitivity analysis
Unsatisfactory*	Sample obscured by debris, lymphocytes or bacteria	Indeterminate	Indeterminate
NMCS	No malignant cells seen	Negative	Negative
Glandular cells	Endometrial glandular cells seen	Negative	Positive
ACUS	Atypical cells of uncertain significance	Negative	Positive
Suspicious	Atypical glandular cells, suspicious for malignancy	Positive	Positive
Adenocarcinoma	Adenocarcinoma malignant cells seen	Positive	Positive
Malignant (other)	Malignant cells of non-endometrial origin	Positive	Positive

*urine according to Paris criteria

Clinical diagnostic pathway

Women will be investigated by transvaginal ultrasound scan (TVS), outpatient hysteroscopy and/or endometrial biopsy according to local clinical diagnostic pathways for the investigation of PMB (Figure 2) (3). All women will have their endometrium measured by TVS. Those with an endometrial thickness <4mm will be considered at low risk of endometrial cancer and alternative diagnoses explored. Women with ≥4mm endometrial thickness will undergo a random endometrial biopsy using a pipelle endometrial sampler. Those with an irregular thickened endometrium, where focal pathology is visualised or suspected, will have an outpatient hysteroscopy and suspicious lesions biopsied under direct vision. Hysteroscopy will be performed under general anaesthesia where outpatient hysteroscopy fails, biopsies are inadequate for diagnostic purposes or uterine instrumentation is poorly tolerated. Endometrial polyps will be resected to allow full histological interpretation. Tissue samples will be formalin-fixed, paraffin embedded, cut into 4µm sections, stained with haematoxylin and eosin (H&E) and cover-slipped as per routine practice. At least one pathologist will review all biopsies; suspicious or abnormal biopsies will be reviewed by two specialist gynaecological pathologists at the cancer centre (St Mary's Hospital) as per routine clinical practice; difficult cases will be reviewed by additional specialist members of the gynaecological pathology team. Hysterectomy specimens will be fixed, cut, sectioned and reviewed according to FIGO-2009 staging criteria (endometrial cancer) and the WHO classification system (atypical hyperplasia) (17, 18).

Reference standard

For peer review only - http://bmjopen.bmj.com/site/about/guidelines.xhtml

The reference standard is histology where endometrial tissue is collected for routine diagnostic and staging purposes. The hysterectomy specimen will be used in preference to the endometrial biopsy, where available within 3 months of endometrial biopsy and where there was no treatment with neoadjuvant chemotherapy or hormone therapy. Histology results will be classified as inadequate, benign, atypical hyperplasia and endometrial cancer (of any histological subtype or grade). For the primary analysis, a histological diagnosis of endometrial cancer will be considered a positive result. Hyperplasia and benign endometrium will be considered a negative result. In secondary analyses, cancers of other urogenital sites (cervix, vagina, ovary, fallopian tube, bladder or kidney) will be considered a positive result. Sensitivity analysis will consider atypical hyperplasia as a positive result. In cases where an endometrial biopsy is not indicated (endometrial thickness <4mm and/or normal hysteroscopy) or fails (inadequate sample), the reference standard will be discharge from diagnostic work up. Clinical follow up of negative women will ensure missed diagnoses of endometrial cancer are minimised. A diagnosis of endometrial cancer within 3 months of initial discharge from diagnostic work up will be considered a false negative result.

Blinding

In most cases, research samples will be collected prior to routine diagnostic work up and sample takers will be blinded to participant cancer outcomes. If the proportion of cases is less than 5% at the midpoint of the study, the cohort will be enriched with endometrial cancer diagnoses by recruiting higher risk women (endometrial thickness >4mm on TVS) and those with proven endometrial cancer prior to hysterectomy. Care will be taken to collect research samples at least 7 days after any diagnostic uterine instrumentation. The cytology team will always be blinded to the cancer status of participants when performing cytological review of urine and vaginal samples. They will have no access to clinical data or results of routine diagnostic tests. Individual participants will not routinely receive their cytology results.

Outcome measures

Primary outcome measure

Sensitivity – the proportion of women with endometrial cancer who test positive by vaginal cytology (true positive rate) and *specificity* – the proportion of women who do not have endometrial cancer who test negative by vaginal cytology (true negative rate). The accuracy of vaginal cytology (index test) will be defined by the results of standard endometrial cancer diagnostic tests (reference standard, Figure 2).

For peer review only - http://bmjopen.bmj.com/site/about/guidelines.xhtml

Secondary outcome measures

Vaginal cytology

1. *Negative predictive value* – the proportion of women with a negative test who do not have endometrial cancer and *positive predictive value* – the proportion of women with a positive test who have endometrial cancer.
2. False positive/ negative rates (and clinical scenarios associated with these)
3. Overall diagnostic accuracy of vaginal cytology for endometrial cancer detection
4. Clinical performance of vaginal cytology for the detection of any cancer of the female genital tract
5. Test acceptability (short questionnaire to compare acceptability of vaginal sampling with standard diagnostic tests)

For peer review only

Urine cytology

1. Sensitivity and specificity of urine cytology for endometrial cancer detection
2. Negative predictive value, positive predictive value, false positive/ negative rates of urine cytology
3. Overall diagnostic accuracy of urine cytology for endometrial cancer detection
4. Clinical performance of urine cytology for the detection of any cancer of the genital or urinary tract
5. Test acceptability (short questionnaire to compare acceptability of urine testing with standard diagnostic tests)

Combined urogenital cytology

1. Sensitivity and specificity of vaginal OR urine cytology for endometrial cancer detection
2. Negative predictive value, positive predictive value, false positive/ negative rates of vaginal OR urine cytology for endometrial cancer detection
3. Overall diagnostic accuracy of vaginal OR urine cytology for endometrial cancer detection
4. Clinical performance of vaginal OR urine cytology for the detection of any cancer of the genital or urinary tract

Handling of discordant results

The accuracy of urogenital cytology will be measured against standard diagnostic tests for endometrial cancer. Concordance of cytology results between observers will be recorded but primary analysis will consider 'suspicious', 'adenocarcinoma' or 'malignant (other)' cytology results

For peer review only - http://bmjopen.bmj.com/site/about/guidelines.xhtml

by consensus opinion to be a positive result. ‘False negative’ results, where standard tests identify endometrial cancer but urogenital cytology does not, will be reviewed to identify possible contributing factors (patient factors, tumour factors or sampling errors). Subject to ongoing consent, women will be re-sampled before they undergo hysterectomy, to determine whether repeat sampling is helpful for missed cases. This will be possible because cytology results will be reported prospectively, in ‘real time’ and on a weekly basis, wherever possible. Cytology review of missed cases will be carried out at a multiheaded microscope by the cytology team. Second slides will be prepared and reviewed where sufficient residual sample allows. ‘False positive’ cases, where urogenital cytology is positive but standard tests are not, will be handled carefully. If the diagnostic pathway (Figure 2) has not been completed, the responsible clinician will be contacted and asked to consider further tests e.g. hysteroscopy or an MRI scan. If the malignant cells identified by cytology could have originated elsewhere, further tests may be warranted (e.g. cystoscopy, colposcopy). Retrospective blinded review of 10% of cases, including test positive, test negative, complex and discrepant cases, will facilitate formal assessment of intra- and inter-observer variability and whether or not there is evidence for a ‘learning curve’ effect.

Assessment of adverse events

Adverse events arising during the study will be recorded and managed in accordance with standard clinical practice.

Data management and monitoring

Data will be managed by a dedicated Project Manager to ensure validity, accuracy and reliability. Data will be handled in accordance with Good Clinical Practice and the Data Protection Act (2018). Participants will be assigned a study specific ID. Written, informed consent will be obtained for all participants and forms stored in local site files within locked filing cabinets. Clinical data will be collected on case report forms (CRFs) and entries verified by inspection against source data. No patient identifiers will be stored in CRFs or on the study database. A sample of CRFs (10% or as per the study risk assessment) will be checked on a regular basis for verification of all entries made. De-identified data will be stored on a study-specific REDCap database. The capture of data on the study database will be checked and verified. Where corrections are required these will carry a full audit trail and justification. The sponsor will periodically audit the study site file, a sample of CRFs, consent forms and source data and accuracy of the study database to ensure satisfactory completion.

Statistical analyses

For peer review only

Statistical analyses will be carried out in R version 3.2.5 (R Development Core Team, Vienna, Austria), and overseen by the trial statistician. A STARD diagram depicting the flow of participants through the study will be presented. This will describe the number of participants who met the inclusion criteria but did not take part and reasons for this. Participants' demographic and clinical characteristics at baseline will be presented by final diagnosis using appropriate descriptive statistics; mean and standard deviation for continuous measures that are approximately symmetric; median and quartiles if the distribution is skewed. Discrete outcomes will be described using both the number and proportion (percentage). The distribution of disease will also be presented for the index test by results of the reference standard.

Analysis of the primary objective will compare the clinical performance of cytology with the reference test. Thus, sensitivity, specificity, negative predictive value, positive predictive value and diagnostic accuracy will be reported together with their corresponding 95% confidence intervals, calculated using the exact binomial method. Where a histology result is not available, endometrial thickness <4mm (histology not indicated) or discharge from diagnostic work up will be used as reference standard. Analysis of the secondary objectives will assess clinical performance of urine test alone as well as combined urogenital cytology for the detection of endometrial cancer in relation to the reference test. The clinical performance of urine and vaginal cytology for the detection of any urological or gynaecological cancer will also be calculated. Secondary analyses will follow the same approach as the primary analysis. Sensitivity analyses will consider the clinical performance of cytology for the detection of atypical hyperplasia and a broader definition of a positive cytology result (including glandular and ACUS results as test positives). Inter-observer agreement between the cytopathologists will be assessed using the Kappa statistic and categorised as poor, fair, moderate and good. McNemar's Chi-squared test will be used to compare urogenital cytology with routine diagnostics. Multivariate logistic regression will evaluate the relationship between positive cytology and endometrial cancer diagnosis whilst adjusting for potentially predictive clinical characteristics like age and BMI. The diagnostic accuracy of urogenital cytology will be compared to individual elements of the standard diagnostic pathway, including transvaginal ultrasound scan, hysteroscopy and endometrial biopsy. Consideration will be given as to where in the current diagnostic pathway for endometrial cancer urogenital cytology fits. Its use as a triage test prior to any other diagnostic work up both alone and in combination with clinical parameters (e.g. age, BMI) will be modelled. We will also consider its usefulness in combination with transvaginal ultrasound scanning at various endometrial thickness cut offs. The acceptability of

For peer review only

urogenital sampling will be compared to transvaginal ultrasound, hysteroscopy and endometrial biopsy (Appendix 1).

Planned secondary use of the data and samples

Beyond the scope of this diagnostic accuracy study, we will use the prospectively collected data to assess the clinical performance of elements of the current diagnostic pathway, alone and in combination, including formal cost effectiveness analyses, if resources permit. We will use the data to validate and compare the clinical performance of several published endometrial cancer risk prediction models, and to develop a novel risk prediction model that includes urogenital cytology. We will report descriptive analyses of our cohort of women with PMB, including the distribution of risk factors and patterns of help seeking behaviour. Residual urine and vaginal samples will be embedded in agar cell blocks or spun down and frozen for future translational research. Agar cell blocks will be used to identify cellular markers by immunohistochemistry that distinguish benign from malignant cells, e.g. proliferation markers (Ki-67, MCM2), which may facilitate their identification using adjunct immunohistochemistry, single cell platforms or flow cytometry. Frozen cell pellets and supernatant fractions will be used to search for novel genomic, metabolomic and proteomic biomarkers.

Ethical approval, research governance, trial sponsorship and registration

This study was approved by the North West - Greater Manchester West Research Ethics Committee (REC reference: 16/NW/0660) and HRA/HRCW on 5th September 2016. Subsequent amendments are detailed in Table 2.

Table 2: Summary of ethical amendments

Protocol	Date	Summary of changes
V3	24.05.2018	Funding and approval secured to expand the study to allow Multicentre recruitment and NIHR portfolio adoption
V4.1	15.10.2018	Additional exclusion criteria added:<br>1. Mirena coil in situ or removed within the last 3 months<br>2. Any other condition that would compromise participant safety or data integrity
V4.3	06.03.2020	Approval to allow recruitment strategy to include proven endometrial cancer cases prior to hysterectomy

For peer review only - http://bmjopen.bmj.com/site/about/guidelines.xhtml

V4.4	16.07.2020	Approval to allow remote consent and data collection, if required, as part of COVID-19 safety mitigation measures

This study was adopted onto the NIHR trial portfolio on 07th August 2018 and is sponsored by Manchester University NHS Foundation Trust. Any planned modifications to the protocol will be approved by the REC before they are adopted by the study. An audit trail of ethical amendments and documentation will be kept to allow monitoring by the research team and external regulatory bodies. The study was registered with an International Standard Randomised Controlled Trial Number (ISRCTN58863784) on 9th August 2018.

Study management

The Study Management Group comprises the Principal Investigator, Project Manager, cytology team, clinical research fellows, research nurses and statistician, who will jointly monitor study conduct and progress. All aspects of the study, and all study personnel, will adhere to the study protocol (version 4.4 or subsequent approved version), Good Clinical Practice and Data Protection principles. Regular team meetings will ensure quick resolution of recruitment issues, study processes and data collection inconsistencies.

Patient and public involvement

The research question was developed in dialogue with patients, carers, members of the public and healthcare professionals. "Which women with abnormal bleeding require specialist referral for investigation?" was ranked the second most important unanswered research question in the James Lind Alliance Womb Cancer Priority Setting Partnership, recognising the need for better diagnostic pathways for endometrial cancer (19). "What simple, non-invasive, painless, cost-effective, and convenient tests can be used to detect cancer early?" was voted the most important research priority for Detecting Cancer Early (8). Urogenital cytology is simple, non-invasive and painless; whether it is effective for the detection of endometrial cancer is the focus of this study. We will disseminate our results through publication in peer-reviewed scientific journals, presentation at conferences and via social media, blogs and charity websites.

DISCUSSION

The DETECT study will establish the diagnostic test accuracy of vaginal and urine samples for endometrial cancer detection by cytology in women with PMB. Urogenital cytology could offer a simple, acceptable, easy to administer test that could be used in primary care as a triage tool for women with unexplained PMB. Cytology positive women could be referred for diagnostic work up

For peer review only

while cytology negative women are quickly reassured without the need for unpleasant, invasive, anxiety-provoking tests, with massive cost-saving implications for the NHS.

For peer review only

For peer review only - http://bmjopen.bmj.com/site/about/guidelines.xhtml

Author Statement: The study was conceived and designed by EJC, who is the study guarantor. All authors contributed to the development and set up of the study. SC is responsible for the day to day running of the study, ethical/regulatory approvals and data management. ERJ, HOF, KN and CEB are responsible for patient recruitment and the collection of data and samples. NN, DS and DR are responsible for the cytological analyses. EJC provides study oversight and clinical guidance for the study. ERJ, SC and EJC drafted the manuscript. All authors contributed to, reviewed and approved the final version of the manuscript.

Funding statement: This work is funded by the Jon Moulton Charity Trust. HOF is supported by a National Institute for Health Research (NIHR) Doctoral Research Fellowship (DRF-2018-11-ST2-054). KN is supported by a Cancer Research UK Manchester Cancer Research Centre Clinical Research Fellowship (C147/A25254) and the Wellcome Trust Manchester Translational Informatics Training Scheme. CEB is supported by a Manchester University NHS Foundation Trust (MFT) Clinical Research Fellowship. EJC is supported by the NIHR Manchester Biomedical Research Centre (IS-BRC-1215-20007).

Data sharing: Fully anonymised data are available from the corresponding author on reasonable request once the study is completed and main findings have been published.

Conflicts of Interest: The authors declare no conflict of interest.

For peer review only

REFERENCES

1. Cancer Research UK. Uterine cancer statistics [Accessed 30th March 2020]. Available from: https://www.cancerresearchuk.org/health-professional/cancer-statistics/statistics-by-cancer-type/uterine-cancer#heading-Zero.

2. Funston G, O'Flynn H, Ryan NAJ, Hamilton W, Crosbie EJ. Recognizing Gynecological Cancer in Primary Care: Risk Factors, Red Flags, and Referrals. Adv Ther. 2018;35(4):577-89.

3. Jones ER, O'Flynn H, Njoku K, Crosbie EJ. Detecting endometrial cancer. The Obstetrician & Gynaecologist. 2021;19 January. doi.org/10.1111/tog.12722

4. Sundar S, Balega J, Crosbie E, Drake A, Edmondson R, Fotopoulou C, et al. BGCS uterine cancer guidelines: Recommendations for practice. Eur J Obstet Gynecol Reprod Biol. 2017;213:71-97.

5. Cooper NA, Barton PM, Breijer M, Caffrey O, Opmeer BC, Timmermans A, et al. Cost-effectiveness of diagnostic strategies for the management of abnormal uterine bleeding (heavy menstrual bleeding and post-menopausal bleeding): a decision analysis. Health technology assessment (Winchester, England). 2014;18(24):1-201, v-vi.

6. Clarke MA, Long BJ, Del Mar Morillo A, Arbyn M, Bakkum-Gamez JN, Wentzensen N. Association of endometrial cancer risk with postmenopausal bleeding in women: A systematic review and meta-analysis. JAMA Internal Medicine. 2018.

7. Walker S, Hyde C, Hamilton W. Risk of uterine cancer in symptomatic women in primary care: case-control study using electronic records. The British journal of general practice : the journal of the Royal College of General Practitioners. 2013;63(614):e643-8.

8. Badrick E, Cresswell K, Ellis P, Renehan AG, Crosbie EJ. Top ten research priorities for detecting cancer early. Lancet Public Health. 2019;4(11):e551.

9. Wang Y, Li L, Douville C, Cohen JD, Yen TT, Kinde I, et al. Evaluation of liquid from the Papanicolaou test and other liquid biopsies for the detection of endometrial and ovarian cancers. Sci Transl Med. 2018;10(433).

10. Kinde I, Bettegowda C, Wang Y, Wu J, Agrawal N, Shih Ie M, et al. Evaluation of DNA from the Papanicolaou test to detect ovarian and endometrial cancers. Sci Transl Med. 2013;5(167):167ra4.

11. Bakkum-Gamez JN, Wentzensen N, Maurer MJ, Hawthorne KM, Voss JS, Kroneman TN, et al. Detection of endometrial cancer via molecular analysis of DNA collected with vaginal tampons. Gynecologic oncology. 2015;137(1):14-22.

For peer review only

12. Frias-Gomez J, Benavente Y, Ponce J, Brunet J, Ibáñez R, Peremiquel-Trillas P, et al. Sensitivity of cervico-vaginal cytology in endometrial carcinoma: A systematic review and meta-analysis. Cancer Cytopathol. 2020.

13. Njoku K, Chiasserini D, Jones ER, Barr CE, O'Flynn H, Whetton AD, Crosbie EJ. Urinary Biomarkers and Their Potential for the Non-Invasive Detection of Endometrial Cancer. Front Oncol. 2020 Nov 3;10:559016.

14. O'Flynn H, Ryan NAJ, Narine N, Shelton D, Rana D, Crosbie EJ. Diagnostic accuracy of cytology for the detection of endometrial cancer in urine and vaginal samples. Nature Communications. 2021. Feb 11;12(1):952.

15. Bossuyt PM, Reitsma JB, Bruns DE, Gatsonis CA, Glasziou PP, Irwig L, et al. STARD 2015: an updated list of essential items for reporting diagnostic accuracy studies. Bmj. 2015;351:h5527.

16. Rovers Medical Devices. Delphi Screener [30th March 2020]. Available from: https://www.roversmedicaldevices.com/cell-sampling-devices/delphi-screener/.

17. FIGO Committee on Gynecologic Oncology. FIGO staging for carcinoma of the vulva, cervix, and corpus uteri. International Journal of Gynecology & Obstetrics. 2014;125(2):97-8.

18. Kurman RJ, Carcangiu ML, Herrington CS, Simon C. World Health Organisation Classification of Tumours of Female Reproductive Organs. International Agency for Research on Cancer 2014;4th Revised ed.

19. Wan YL, Beverley-Stevenson R, Carlisle D, Clarke S, Edmondson RJ, Glover S, et al. Working together to shape the endometrial cancer research agenda: The top ten unanswered research questions. Gynecologic oncology. 2016;143(2):287-93.

For peer review only

Figure legends

Figure 1: Study schema illustrating the flow of participants through the study, interventions and evaluations

Figure 2: Diagnostic pathway for women referred to secondary care for the urgent investigation of unexplained PMB

For peer review only

For peer review only - http://bmjopen.bmj.com/site/about/guidelines.xhtml

Figure 1. Study schema

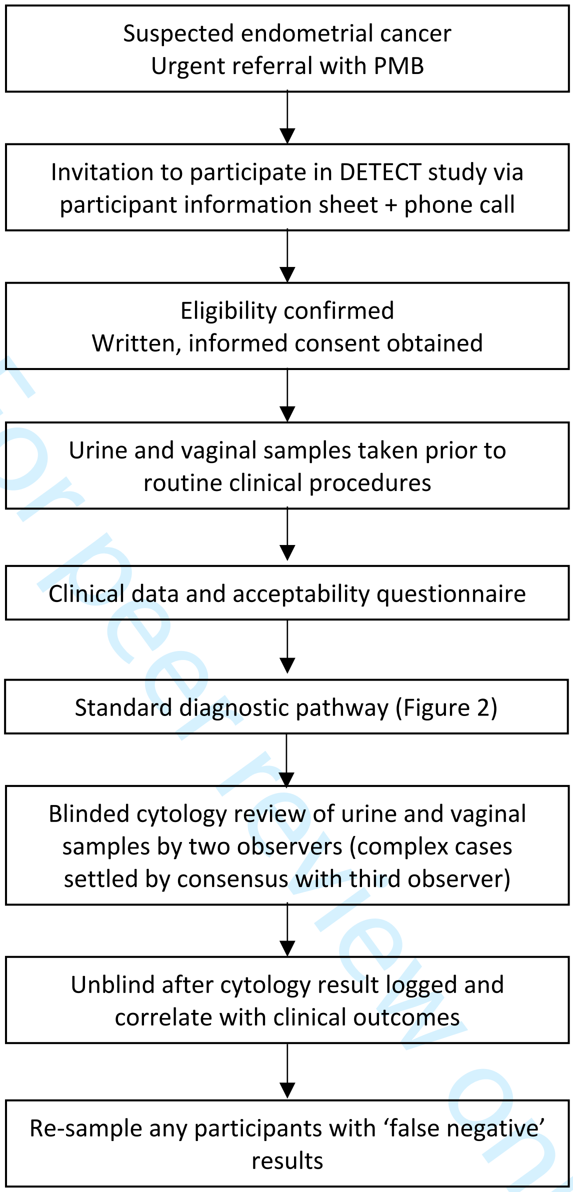

For peer review only

Figure 2: Diagnostic pathway

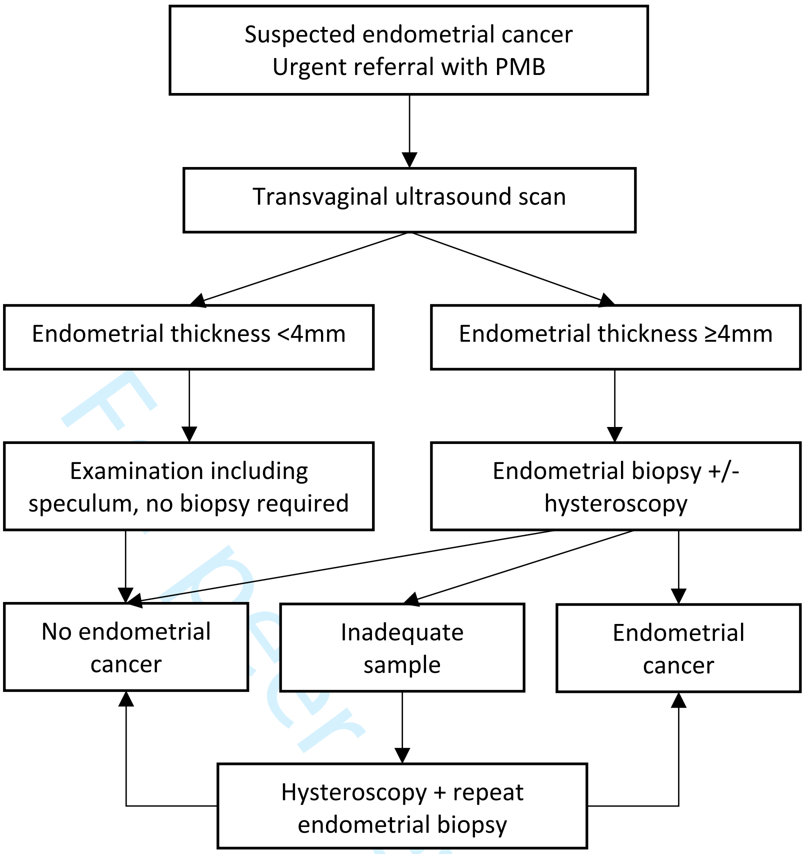

For peer review only

Appendix 1. Acceptability questionnaire

How would you describe your education level?

Primary School (Up to age 11)	☐
Secondary school (Up to age 16)	☐
Higher Education or above (A Levels/ University)	☐

	Yes	No
**Have you used any vaginal creams/ pessaries before?**	☐	☐
**Have you ever used tampons?**	☐	☐
**Have you given a urine sample before?**	☐	☐
**Have you ever used a home pregnancy test?**	☐	☐

Have you been sexually active?

Within the last month	☐
Within the last year	☐
More than a year/ not sexually active	☐
Would rather not say	☐

How would you describe the amount of discomfort experienced during the vaginal washing?

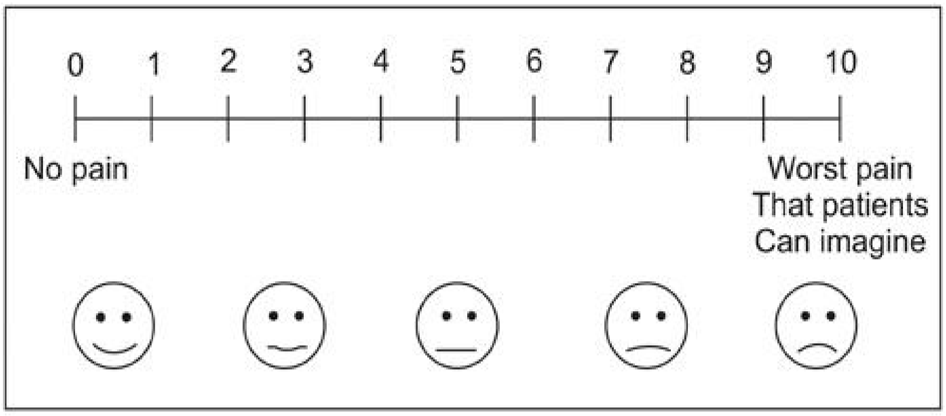

For peer review only

How would you describe the amount of discomfort experienced during pelvic examination?

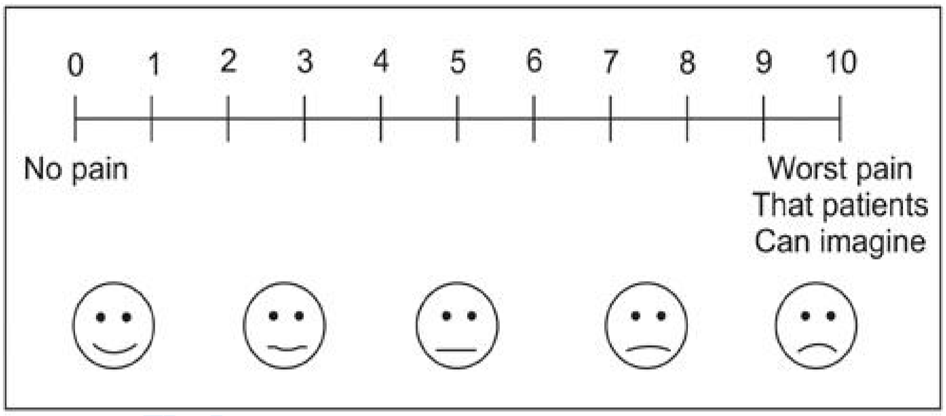

How would you describe the amount of discomfort experienced during ultrasound scan?

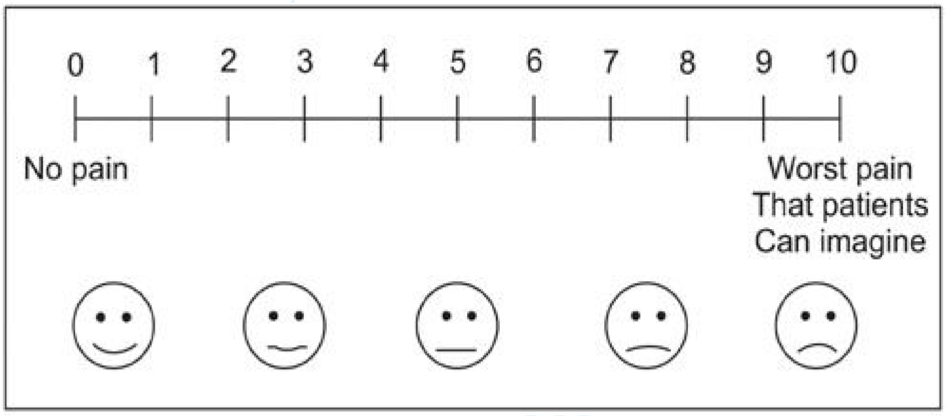

How would you describe the amount of discomfort experienced during hysteroscopy?

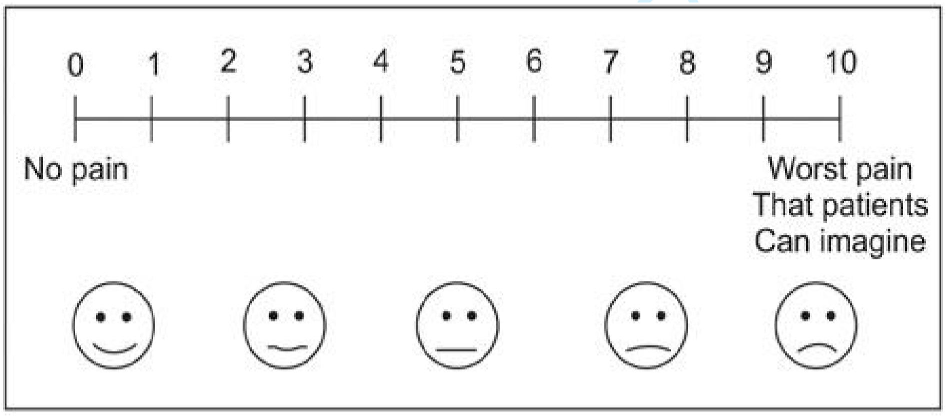

For peer review only - http://bmjopen.bmj.com/site/about/guidelines.xhtml

How would you describe the amount of discomfort experienced during endometrial biopsy sampling?

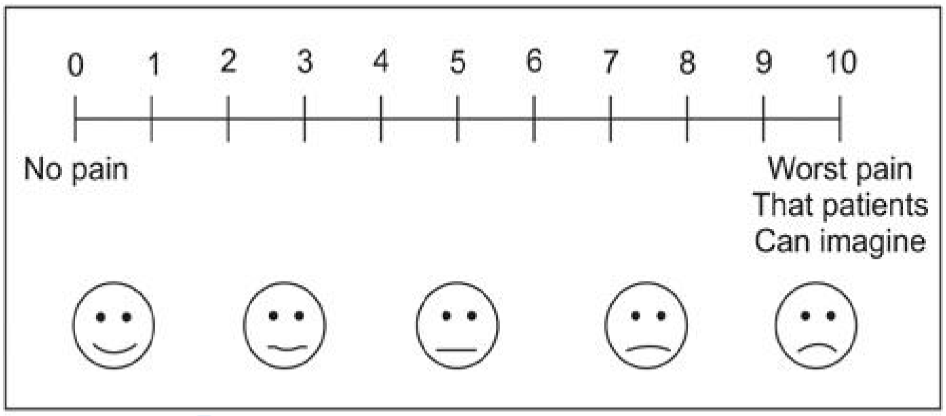

If needed how likely are you to have the following investigations again?

	**Definitely not**	**Probably not**	**Not sure**	**Probably**	**Definitely**
Ultrasound scan	☐	☐	☐	☐	☐
Vaginal washing	☐	☐	☐	☐	☐
Urine sample	☐	☐	☐	☐	☐
Pelvic examination	☐	☐	☐	☐	☐
Hysteroscopy	☐	☐	☐	☐	☐
Endometrial biopsy	☐	☐	☐	☐	☐

or peer review only

For peer review only - http://bmjopen.bmj.com/site/about/guidelines.xhtml

BMJ Open

DEveloping Tests for Endometrial Cancer deTection (DETECT): Protocol for a diagnostic accuracy study of urine and vaginal samples for the detection of endometrial cancer by cytology in women with postmenopausal bleeding

Journal:	*BMJ Open*
Manuscript ID	bmjopen-2021-050755.R1
Article Type:	Protocol
Date Submitted by the Author:	15-Jun-2021

Complete List of Authors:	Jones, Eleanor ; The University of Manchester, Division of Cancer Sciences<br>Carter, Suzanne; The University of Manchester<br>O'Flynn, Helena; The University of Manchester<br>Njoku, Kelechi; The University of Manchester<br>Barr, Chloe ; The University of Manchester, Department of Obstetrics and Gynaecology<br>Narine, Nadira; Manchester University NHS Foundation Trust<br>Shelton, David; Manchester University NHS Foundation Trust<br>Rana, Durgesh; Manchester University NHS Foundation Trust<br>Crosbie, Emma; The University of Manchester

**Primary Subject Heading**:	Obstetrics and gynaecology
Secondary Subject Heading:	Oncology
Keywords:	Gynaecological oncology < GYNAECOLOGY, CYTOPATHOLOGY, PRIMARY CARE

SCHOLARONE™
Manuscripts

I, the Submitting Author has the right to grant and does grant on behalf of all authors of the Work (as defined in the below author licence), an exclusive licence and/or a non-exclusive licence for contributions from authors who are: i) UK Crown employees; ii) where BMJ has agreed a CC-BY licence shall apply, and/or iii) in accordance with the terms applicable for US Federal Government officers or employees acting as part of their official duties; on a worldwide, perpetual, irrevocable, royalty-free basis to BMJ Publishing Group Ltd ("BMJ") its licensees and where the relevant Journal is co-owned by BMJ to the co-owners of the Journal, to publish the Work in this journal and any other BMJ products and to exploit all rights, as set out in our licence.

The Submitting Author accepts and understands that any supply made under these terms is made by BMJ to the Submitting Author unless you are acting as an employee on behalf of your employer or a postgraduate student of an affiliated institution which is paying any applicable article publishing charge ("APC") for Open Access articles. Where the Submitting Author wishes to make the Work available on an Open Access basis (and intends to pay the relevant APC), the terms of reuse of such Open Access shall be governed by a Creative Commons licence – details of these licences and which Creative Commons licence will apply to this Work are set out in our licence referred to above.

Other than as permitted in any relevant BMJ Author's Self Archiving Policies, I confirm this Work has not been accepted for publication elsewhere, is not being considered for publication elsewhere and does not duplicate material already published. I confirm all authors consent to publication of this Work and authorise the granting of this licence.

For peer review only

DEveloping Tests for Endometrial Cancer deTection (DETECT): Protocol for a diagnostic accuracy study of urine and vaginal samples for the detection of endometrial cancer by cytology in women with postmenopausal bleeding

Eleanor R. Jones[1], Suzanne Carter[1], Helena O'Flynn[1], Kelechi Njoku[1], Chloe E. Barr[1,2], Nadira Narine[3], David Shelton[3], Durgesh Rana[3], Emma J. Crosbie[1,2]*

[1]Gynaecological Oncology Research Group, Division of Cancer Sciences, Faculty of Biology, Medicine and Health, University of Manchester, Manchester, UK

[2]Department of Obstetrics and Gynaecology, St Mary's Hospital, Manchester University NHS Foundation Trust, Manchester Academic Health Science Centre, Manchester, UK

[3]Manchester Cytology Centre, Manchester University Hospitals NHS Foundation Trust, Manchester Academic Health Science Centre, Manchester, UK

*Corresponding author: Professor Emma Crosbie, Division of Cancer Sciences, University of Manchester, School of Medical Sciences, Faculty of Biology, Medicine and Health, 5th Floor Research, St Mary's Hospital, Oxford Road, Manchester M13 9WL. Email: emma.crosbie@manchester.ac.uk. Phone. 0161 701 6942

International Standard Randomised Controlled Trial Number: ISRCTN58863784

Key words: endometrial cancer, postmenopausal bleeding, early detection, non-invasive sampling, diagnostic tool, cytology

For peer review only

Abstract

Introduction: Postmenopausal bleeding (PMB), the red flag symptom for endometrial cancer, triggers urgent investigation by transvaginal ultrasound scan, hysteroscopy and/or endometrial biopsy. These investigations are costly, invasive and often painful or distressing for women. In a pilot study, we found that voided urine and non-invasive vaginal samples from women with endometrial cancer contain malignant cells that can be identified by cytology. The aim of the DETECT study is to determine the diagnostic test accuracy of urine and vaginal cytology for endometrial cancer detection in women with PMB.

Methods and analysis: This is a multi-centre diagnostic accuracy study of women referred to secondary care with PMB. Eligible women will be asked to provide a self-collected voided urine sample and a vaginal sample collected with a Delphi screener before routine clinical procedures. Pairs of specialist cytologists, blinded to participant cancer status, will assess and classify samples independently, with differences settled by consensus review or involving a third cytologist. Results will be compared with clinical outcomes from standard diagnostic tests. A sample size of 2,000 women will have 80% power to establish a sensitivity of vaginal samples for endometrial cancer detection by cytology of ≥85% ±7%, assuming 5% endometrial cancer prevalence. The primary objective is to determine the diagnostic accuracy of vaginal samples for endometrial cancer detection by cytology. Secondary objectives include the diagnostic accuracy of urine and combined urogenital cytology, and the acceptability of urine and vaginal sampling to women.

Ethics and dissemination: This study has been approved by the North West-Greater Manchester West Research Ethics Committee (16/NW/0660) and the Health Research Authority. Results will be disseminated through publication in peer-reviewed scientific journals, presentation at conferences and via charity websites.

For peer review only

Strengths and limitations of this study

- This is a prospective evaluation of a novel, non-invasive endometrial cancer detection tool that could transform diagnostic pathways for women with PMB
- Samples will be taken prior to routine clinical procedures to avoid inadvertent contamination of samples by iatrogenically-dislodged endometrial cells
- Cytologists are blinded to participant cancer status until they provide their consensus report
- Passive follow up of participants will ensure missed cancer diagnoses are minimised
- Recruitment is limited to women with PMB and results may not be applicable to premenopausal women or those with atypical presentations of endometrial cancer

For peer review only

For peer review only - http://bmjopen.bmj.com/site/about/guidelines.xhtml

INTRODUCTION

In the UK, over 9,000 women are diagnosed with endometrial cancer every year (1). The red flag symptom for endometrial cancer is postmenopausal bleeding (PMB) (2). A woman with PMB is referred by her GP on the urgent 'suspected cancer' pathway to a rapid access gynaecology clinic, where she is offered a series of invasive, unpleasant and often painful tests to rule out endometrial cancer (3). These include a transvaginal ultrasound scan, an outpatient hysteroscopy and an endometrial biopsy (4). Together, these tests cost the NHS around £750/woman (5). PMB is extremely common and only 5-10% of women with PMB are ultimately diagnosed with endometrial cancer (6, 7). Indeed, it has been estimated that 5% of all GP referrals to gynaecology, as many as 150,000 women per year in the UK alone, relate to PMB (5). A simple, non-invasive test deployed in primary care to target those at risk of endometrial cancer for invasive testing, whilst safely reassuring the vast majority of healthy women, could transform diagnostic pathways for endometrial cancer. In the UK, it would save thousands of women every year from the psychological and physical sequelae of invasive tests and create substantial cost savings for the NHS (potential saving in excess of £100M/year).

The development of novel non-invasive detection tools was voted the most important research priority for detecting cancer early in the recently completed James Lind Alliance Priority Setting Partnership (8). In endometrial cancer, the anatomical continuity between the uterus and the lower genital tract facilitates the collection of shed tumour cells using non-invasive sampling methodologies (9-13). We found that shed tumour cells can be collected from the vagina by gentle lavage using the Delphi screener, and from voided urine samples, which are inevitably contaminated by endometrial debris in women with uterine bleeding (14). These cells can be distinguished from normal squamous, urothelial and glandular cells of the urogenital tract by cytology, although certain benign mimics (e.g. polyps, atrophy) can cause difficulties with interpretation. In our pilot study of 113 women with unexplained PMB, urine and/or vaginal cytology showed 100% sensitivity and 100% negative predictive value for endometrial cancer detection; it identified all 7 cancers (4 endometrial, 1 cervical, 1 ovarian and 1 bladder) for a 11% false positive rate. Furthermore, mean pain scores were significantly lower for vaginal sampling (1.61, SD 2.04) than for diagnostic hysteroscopy (4.28, SD 2.61, $p<0.001$) and endometrial biopsy (4.88, SD 3.49, $p<0.001$), respectively (14). Thus urogenital cytology has considerable potential as a well tolerated 'rule out' test to enable quick reassurance for most women who present to primary care with PMB, and urgent referral for those who test positive. To confirm its clinical utility for endometrial cancer detection, urine and vaginal cytology must now be tested in a large prospective study of women undergoing investigation for PMB. The aim of the DEveloping Tests for Endometrial Cancer deTection (DETECT) study is to estimate the diagnostic

For peer review only - http://bmjopen.bmj.com/site/about/guidelines.xhtml

accuracy of urine and vaginal samples for endometrial cancer detection by cytology in women with PMB.

METHODS

This protocol is reported in accordance with Standards for Reporting of Diagnostic Accuracy Studies (STARD) guidelines (15).

Study design

DETECT is a prospective multicentre diagnostic accuracy study of urine and vaginal cytology for endometrial cancer detection in women with PMB (Study schema, Figure 1).

Participants and recruitment

Consecutive women will be recruited from gynaecology clinics at seven hospital sites across the North West of England: St Mary's Hospital, Trafford General Hospital, Wythenshawe Hospital, Royal Oldham Hospital, North Manchester General Hospital, Fairfield General Hospital and Tameside Hospital.

Inclusion criteria

1. Women who have been referred to secondary care for investigation of PMB
2. Written, informed consent to participate

Exclusion criteria

1. Abnormal bleeding before the menopause
2. Previous treatment for endometrial cancer
3. Previous hysterectomy
4. Mirena coil in situ or removed within the last 3 months
5. Any other condition that would compromise participant safety or data integrity

PMB will be defined as vaginal bleeding that occurs more than 12 months after menstruation has stopped due to menopause.

Participant withdrawal

Participants may withdraw from the study at their own request or at the discretion of the Investigator. Withdrawal from the study will not affect patient care.

For peer review only

Sample size

With a sample size of ~2,000 women, and an endometrial cancer prevalence of 5% (5), there will be approximately 1,900 women without endometrial cancer and 100 women with endometrial cancer. The study will have 80% power to determine the sensitivity of the test at ≥85% +/-7% and the specificity of the test at ≥85% +/-2%. At 85% specificity, around 1,630 women will have a negative test result, giving the test a negative predictive value (NPV) of 99.1% (98.5%, 99.4%). These estimates originate from the pilot study, where sensitivity and specificity were both >85%. The prevalence of endometrial cancer will determine the final sample size. If the prevalence of endometrial cancer is greater than 5%, fewer than 2,000 women will be needed; if it is less than 5%, more women will be needed to determine the sensitivity of the test at ≥ 85% +/-7%.

Study duration

In the pilot study, 90% of eligible women agreed to participate. Women will be recruited between September 2018 and September 2021 with clinical outcome data collected until March 2022. To achieve the recruitment target of ~2,000 women, approximately 20 women will be recruited per week across the seven hospital sites. A temporary pause on recruitment to the study was initiated in March 2020 because of the COVID-19 pandemic. Recruitment recommenced in June 2020 but with COVID-19 restrictions in place, meaning that recruitment rate is slower than pre-pandemic (approximately 10 women per week across recruitment sites).

Invitational procedure

Eligible women will be identified from referral letters and clinic lists by hospital clinical staff. A letter inviting participation and a detailed participant information sheet (PIS) will be sent by post to their home address. Potential participants will have the opportunity to discuss the study over the telephone with members of the study research team prior to their appointment. Due to the urgent nature of referrals, some women are offered short notice clinical appointments by telephone. Where there is insufficient time for invitational material to be received by post, women will be informed about the study via telephone and/or invited to participate on arrival at the clinic. Women will be invited to read the PIS and ask questions about the study before providing written, informed consent to participate.

Baseline clinical data

The following demographic and baseline clinical data will be obtained: age, ethnicity, socioeconomic status, education level, body mass index (BMI), smoking status, parity, age of menopause, use of

For peer review only

contraceptives, hormone replacement therapy (HRT) or tamoxifen, history of hypertension, hypercholesterolaemia, diabetes mellitus, polycystic ovary syndrome, thyroid disease, coagulopathy, Lynch syndrome, cervical screening history, endometrial hyperplasia, and personal or family history of cancer. Women will be asked in detail about their help seeking behaviour and the onset, duration and extent of their PMB.

Index tests

The timing of urine and vaginal sampling is important to ensure validity of the results. Both research samples will be taken before any routine clinical procedures to avoid inadvertent contamination with iatrogenically-dislodged endometrial cells (Study schema, Figure 1). Of the two research samples, urine will be collected first because vaginal sampling will remove naturally shed uterine debris from the lower genital tract and affect interpretation of the results.

Urine samples

Using both written and verbal instructions, we will ask women to bring to clinic a first catch sample of their first urinary void of the day, collected in a sterile pot. A second voided urine sample will be collected by the participant upon arrival at the clinic, before any other research or clinical procedures are carried out. This will ensure that every woman has at least one satisfactory urine sample available for analysis.

Vaginal sample

The vaginal sample will be taken by the research practitioner using a Delphi screener (Rovers Medical Devices, OSS, Netherlands) (16) according to the following protocol, with the participant in the supine position, knees bent and legs apart. The Delphi screener is inserted into the posterior fornix of the vagina and 3ml saline expelled from its reservoir by depressing the plunger for three seconds. The sample is then collected by suction following release of the plunger whilst slowly rotating and withdrawing the device. A dry pot at the introitus collects any residual fluid. Additional samples are obtained by re-filling the reservoir with saline and repeating the steps above until clear fluid is obtained (maximum of three times).

Sample handling

Urine samples will be tested for haematuria by dipstick. Urine and vaginal samples will be fixed with equal volumes of BD CytoRich™ Red Preservative (Becton, Dickinson and Company, USA) to preserve cellular integrity, prevent degradation and inhibit bacterial overgrowth. Samples will be

For peer review only - http://bmjopen.bmj.com/site/about/guidelines.xhtml

sent to the Manchester Cytology Centre at the Manchester University NHS Foundation Trust (MFT). Samples will be anonymised and labelled with sample type (urine or vaginal fluid) and a unique study identifier (study ID) to prevent accidental unblinding of the cytopathology team. With the exception of whether the participant is taking exogenous hormones, all clinical data will be withheld from the cytology team until consensus results are received. Second urine samples will be fixed with BD CytoRich^{TM} Red and stored at 4°C until the first urine sample has undergone cytological assessment. This second sample will undergo cytological review if the first urine sample is inadequate for analysis or post hoc, if the first urine sample is false negative for endometrial cancer detection. Residual samples will be stored in the MFT Biobank for future biomarker discovery work. Samples will either be embedded in agar cell blocks to preserve cellular integrity for future immunohistochemistry or centrifuged to pellet the cellular material. The resulting pellet plus aliquots of the supernatant will be stored at -80°C.

Cytological assessment

Samples will be centrifuged at 3000RPM for 5 minutes, supernatant decanted and the pellet resuspended in 6mls CytoRich^{TM} Red. After 1 hour, the fixed sample will be centrifuged at 1500RPM for 10 minutes, supernatant decanted and the remaining pellet prepared into a liquid based cytology Papanicolaou stained slide using the BD prepstain (Becton Dickinson UK Limited) according to the manufacturer's instructions. The stained slide will be dehydrated in two changes of industrial methylated spirits, cleared in two changes of xylene and cover-slipped. Two observers, a consultant cytopathologist and a consultant Biomedical Scientist (BMS), will review each slide independently and record their results. A second consultant cytopathologist will review any discrepant cases, which will be settled by consensus review at a multi-headed microscope. Final cytology results will be logged under the unique study ID on the study database. Intra- and inter-observer variability will be determined by blinded, independent review of a random selection of anonymised test positive, test negative, complex and discrepant cases at the end of the study, and reported separately.

Classification of cytology results

Cytology slides will be reported according to the classification system shown in Table 1. For the primary analysis, atypical cells of uncertain significance (ACUS), suspicious, adenocarcinoma or malignant (other) cytology results will be considered positive. Glandular cells and no malignant cells seen (NMCS) will be considered negative. Unsatisfactory results will not be classified as either positive or negative and the participant will be invited to provide a second sample for

For peer review only - http://bmjopen.bmj.com/site/about/guidelines.xhtml

cytological analysis. Secondary analysis will include cancers of other urogenital sites (cervix, vagina, ovary, fallopian tube, bladder or kidney). Sensitivity analysis will consider the diagnostic performance of positive urogenital cytology that includes glandular results as potentially malignant findings.

Table 1: Cytological classification

Cytology result	Cytological findings	Primary analysis	Sensitivity analysis
Unsatisfactory*	Sample obscured by debris, lymphocytes or bacteria	Indeterminate	Indeterminate
NMCS	No malignant cells seen	Negative	Negative
Glandular cells	Endometrial glandular cells seen	Negative	Positive
ACUS	Atypical cells of uncertain significance	Positive	Positive
Suspicious	Atypical glandular cells, suspicious for malignancy	Positive	Positive
Adenocarcinoma	Adenocarcinoma malignant cells seen	Positive	Positive
Malignant (other)	Malignant cells of non-endometrial origin	Positive	Positive

*urine according to Paris criteria

Clinical diagnostic pathway

Women will be investigated by transvaginal ultrasound scan (TVS), outpatient hysteroscopy and/or endometrial biopsy according to local clinical diagnostic pathways for the investigation of PMB (Figure 2) (3). All women will have their endometrium measured by TVS. Those with an endometrial thickness <4mm will be considered at low risk of endometrial cancer and alternative diagnoses explored. Women with ≥4mm endometrial thickness will undergo an endometrial biopsy using a pipelle endometrial sampler. Those with an irregular thickened endometrium, where focal pathology is visualised or suspected, will have an outpatient hysteroscopy and suspicious lesions biopsied under direct vision. Hysteroscopy will be performed under general anaesthesia where outpatient hysteroscopy fails, biopsies are inadequate for diagnostic purposes or uterine instrumentation is poorly tolerated. Endometrial polyps will be resected to allow full histological interpretation. Tissue samples will be formalin-fixed, paraffin embedded, cut into 4μm sections, stained with haematoxylin and eosin (H&E) and cover-slipped as per routine practice. At least one pathologist will review all biopsies; suspicious or abnormal biopsies will be reviewed by two specialist gynaecological pathologists at the cancer centre (St Mary's Hospital) as per routine clinical practice; difficult cases will be reviewed by additional specialist members of the gynaecological pathology team. Hysterectomy specimens will be fixed, cut, sectioned and reviewed according to FIGO-2009 staging criteria (endometrial cancer) and the WHO classification system (atypical hyperplasia) (17, 18).

For peer review only - http://bmjopen.bmj.com/site/about/guidelines.xhtml

Reference standard

The reference standard is histology where endometrial tissue is collected for routine diagnostic and staging purposes. The hysterectomy specimen will be used in preference to the endometrial biopsy, where available within 3 months of endometrial biopsy and where there was no treatment with neoadjuvant chemotherapy or hormone therapy. Histology results will be classified as inadequate, benign, atypical hyperplasia and endometrial cancer (of any histological subtype or grade). For the primary analysis, a histological diagnosis of endometrial cancer will be considered a positive result. We will report overall results as well as a breakdown by histological subtype and stage of disease. Hyperplasia and benign endometrium will be considered a negative result. In secondary analyses, cancers of other urogenital sites (cervix, vagina, ovary, fallopian tube, bladder or kidney) will be considered a positive result. We will record cases of atypical hyperplasia and their cytological interpretation. In cases where an endometrial biopsy is not indicated (endometrial thickness <4mm and/or normal hysteroscopy) or fails (inadequate sample), the reference standard will be discharge from diagnostic work up. Passive clinical follow up of negative women will ensure missed diagnoses of endometrial cancer are minimised. This will involve monitoring for any subsequent re-referrals to our service. A diagnosis of endometrial cancer within 3 months of initial discharge from diagnostic work up will be considered a positive clinical result.

Blinding

In most cases, research samples will be collected prior to routine diagnostic work up and sample takers will be blinded to participant cancer outcomes. If the proportion of cases is less than 5% at the midpoint of the study, the cohort will be enriched with endometrial cancer diagnoses by recruiting higher risk women (endometrial thickness >4mm on TVS) and those with proven endometrial cancer prior to hysterectomy. Care will be taken to collect research samples at least 7 days after any diagnostic uterine instrumentation. The cytology team will always be blinded to the cancer status of participants when performing cytological review of urine and vaginal samples. They will have no access to clinical data or results of routine diagnostic tests. Individual participants will not routinely receive their cytology results.

Outcome measures

Primary outcome measure

Sensitivity – the proportion of women with endometrial cancer who test positive by vaginal cytology (true positive rate) and *specificity* – the proportion of women who do not have endometrial cancer who test negative by vaginal cytology (true negative rate). The accuracy of

For peer review only - http://bmjopen.bmj.com/site/about/guidelines.xhtml

vaginal cytology (index test) will be defined by the results of standard endometrial cancer diagnostic tests (reference standard, Figure 2).

Secondary outcome measures

Vaginal cytology

1. *Negative predictive value* – the proportion of women with a negative test who do not have endometrial cancer and *positive predictive value* – the proportion of women with a positive test who have endometrial cancer.
2. False positive/ negative rates (and clinical scenarios associated with these)
3. Overall diagnostic accuracy of vaginal cytology for endometrial cancer detection
4. Clinical performance of vaginal cytology for the detection of any cancer involving the female genital tract
5. Test acceptability (short questionnaire to compare acceptability of vaginal sampling with standard diagnostic tests in a proportion of participants, eg 5-10%)

Urine cytology

1. Sensitivity and specificity of urine cytology for endometrial cancer detection
2. Negative predictive value, positive predictive value, false positive/ negative rates of urine cytology
3. Overall diagnostic accuracy of urine cytology for endometrial cancer detection
4. Clinical performance of urine cytology for the detection of any cancer involving the genital or urinary tract
5. Test acceptability (short questionnaire to compare acceptability of urine testing with standard diagnostic tests)

Combined urogenital cytology

1. Sensitivity and specificity of vaginal OR urine cytology for endometrial cancer detection
2. Negative predictive value, positive predictive value, false positive/ negative rates of vaginal OR urine cytology for endometrial cancer detection
3. Overall diagnostic accuracy of vaginal OR urine cytology for endometrial cancer detection
4. Clinical performance of vaginal OR urine cytology for the detection of any cancer involving the genital or urinary tract

Handling of discordant results

For peer review only

The accuracy of urogenital cytology will be measured against standard diagnostic tests for endometrial cancer. Concordance of cytology results between observers will be recorded but primary analysis will consider 'ACUS', 'suspicious', 'adenocarcinoma' or 'malignant (other)' cytology results by consensus opinion to be a positive result. 'False negative' results, where standard tests identify endometrial cancer but urogenital cytology does not, will be reviewed to identify possible contributing factors (patient factors, tumour factors or test errors). Subject to ongoing consent, women will be re-sampled before they undergo hysterectomy, to determine whether repeat sampling is helpful for missed cases. This will be possible because cytology results will be reported prospectively, in 'real time' and on a weekly basis, wherever possible. Cytology review of missed cases will be carried out at a multiheaded microscope by the cytology team. Second +/- further slides will be prepared and reviewed where sufficient residual sample allows. 'False positive' cases, where urogenital cytology is positive but standard tests are not, will be handled carefully. If the diagnostic pathway (Figure 2) has not been completed, the responsible clinician will be contacted and asked to consider further tests e.g. hysteroscopy or an MRI scan. If the malignant cells identified by cytology could have originated elsewhere, further tests may be warranted (e.g. cystoscopy, colposcopy). Retrospective blinded review of 10% of cases, including test positive, test negative, complex and discrepant cases, will facilitate formal assessment of intra- and inter-observer variability and whether or not there is evidence for a 'learning curve' effect.

Assessment of adverse events

Adverse events arising during the study will be recorded and managed in accordance with standard clinical practice.

Data management and monitoring

Data will be managed by a dedicated Project Manager to ensure validity, accuracy and reliability. Data will be handled in accordance with Good Clinical Practice and the Data Protection Act (2018). Participants will be assigned a study specific ID. Written, informed consent will be obtained for all participants and forms stored in local site files within locked filing cabinets. Clinical data will be collected on case report forms (CRFs) and entries verified by inspection against source data. No patient identifiers will be stored in CRFs or on the study database. A sample of CRFs (10% or as per the study risk assessment) will be checked on a regular basis for verification of all entries made. De-identified data will be stored on a study-specific REDCap database. The capture of data on the study database will be checked and verified. Where corrections are required these will carry a full audit

For peer review only

For peer review only - http://bmjopen.bmj.com/site/about/guidelines.xhtml

trail and justification. The sponsor will periodically audit the study site file, a sample of CRFs, consent forms and source data and accuracy of the study database to ensure satisfactory completion.

Statistical analyses

Statistical analyses will be carried out in R version 3.2.5 (R Development Core Team, Vienna, Austria), and overseen by the trial statistician. A STARD diagram depicting the flow of participants through the study will be presented. This will describe the number of participants who met the inclusion criteria but did not take part and reasons for this. Participants' demographic and clinical characteristics at baseline will be presented by final diagnosis using appropriate descriptive statistics; mean and standard deviation for continuous measures that are approximately symmetric; median and quartiles if the distribution is skewed. Discrete outcomes will be described using both the number and proportion (percentage). The distribution of disease will also be presented for the index test by results of the reference standard.

Analysis of the primary objective will compare the clinical performance of cytology with the reference test. Thus, sensitivity, specificity, negative predictive value, positive predictive value and diagnostic accuracy will be reported together with their corresponding 95% confidence intervals, calculated using the exact binomial method. Where a histology result is not available, endometrial thickness <4mm (histology not indicated) or discharge from diagnostic work up will be used as reference standard. Analysis of the secondary objectives will assess clinical performance of urine test alone as well as combined urogenital cytology for the detection of endometrial cancer in relation to the reference test. The clinical performance of urine and vaginal cytology for the detection of any cancer affecting the urological or gynaecological organs will also be calculated. Secondary analyses will follow the same approach as the primary analysis. Sensitivity analyses will consider the clinical performance of cytology with a broader definition of a positive cytology result (including glandular results as test positives). Inter-observer agreement between the cytopathologists will be assessed using the Kappa statistic and categorised as poor, fair, moderate and good. McNemar's Chi-squared test will be used to compare urogenital cytology with routine diagnostics. Multivariate logistic regression will evaluate the relationship between positive cytology and endometrial cancer diagnosis whilst adjusting for potentially predictive clinical characteristics like age and BMI. The diagnostic accuracy of urogenital cytology will be compared to individual elements of the standard diagnostic pathway, including transvaginal ultrasound scan, hysteroscopy and endometrial biopsy. Consideration will be given as to where in the current diagnostic pathway for endometrial cancer urogenital cytology fits. Its use as a triage test prior to any other diagnostic

For peer review only - http://bmjopen.bmj.com/site/about/guidelines.xhtml

work up both alone and in combination with clinical parameters (e.g. age, BMI) will be modelled. We will also consider its usefulness in combination with transvaginal ultrasound scanning at various endometrial thickness cut offs. The acceptability of urogenital sampling will be compared to transvaginal ultrasound, hysteroscopy and endometrial biopsy (Appendix 1).

Planned secondary use of the data and samples

Beyond the scope of this diagnostic accuracy study, we will use the prospectively collected data to assess the clinical performance of elements of the current diagnostic pathway, alone and in combination, including formal cost effectiveness analyses, if resources permit. We will use the data to validate and compare the clinical performance of several published endometrial cancer risk prediction models, and to develop a novel risk prediction model that includes urogenital cytology. We will report descriptive analyses of our cohort of women with PMB, including the distribution of risk factors and patterns of help seeking behaviour. Residual urine and vaginal samples will be embedded in agar cell blocks or spun down and frozen for future translational research. Agar cell blocks will be used to identify cellular markers by immunohistochemistry that distinguish benign from malignant cells, e.g. proliferation markers (Ki-67, MCM2), which may facilitate their identification using adjunct immunohistochemistry, single cell platforms or flow cytometry. Frozen cell pellets and supernatant fractions will be used to search for novel genomic, metabolomic and proteomic biomarkers.

Ethics and dissemination

Ethical approval, research governance, trial sponsorship and registration

This study was approved by the North West - Greater Manchester West Research Ethics Committee (REC reference: 16/NW/0660) and HRA/HRCW on 5th September 2016. Subsequent amendments are detailed in Table 2.

Table 2: Summary of ethical amendments

Protocol	Date	Summary of changes
V3	24.05.2018	Funding and approval secured to expand the study to allow Multicentre recruitment and NIHR portfolio adoption
V4.1	15.10.2018	Additional exclusion criteria added:<br>1. Mirena coil in situ or removed within the last 3 months<br>2. Any other condition that would compromise participant safety or data

For peer review only

		integrity
V4.3	06.03.2020	Approval to allow recruitment strategy to include proven endometrial cancer cases prior to hysterectomy
V4.4	16.07.2020	Approval to allow remote consent and data collection, if required, as part of COVID-19 safety mitigation measures

This study was adopted onto the NIHR trial portfolio on 07th August 2018 and is sponsored by Manchester University NHS Foundation Trust. Any planned modifications to the protocol will be approved by the REC before they are adopted by the study. An audit trail of ethical amendments and documentation will be kept to allow monitoring by the research team and external regulatory bodies. The study was registered with an International Standard Randomised Controlled Trial Number (ISRCTN58863784) on 9th August 2018.

Study management

The Study Management Group comprises the Principal Investigator, Project Manager, cytology team, clinical research fellows, research nurses and statistician, who will jointly monitor study conduct and progress. All aspects of the study, and all study personnel, will adhere to the study protocol (version 4.4 or subsequent approved version), Good Clinical Practice and Data Protection principles. Regular team meetings will ensure quick resolution of recruitment issues, study processes and data collection inconsistencies.

Patient and public involvement

The research question was developed in dialogue with patients, carers, members of the public and healthcare professionals. “Which women with abnormal bleeding require specialist referral for investigation?” was ranked the second most important unanswered research question in the James Lind Alliance Womb Cancer Priority Setting Partnership, recognising the need for better diagnostic pathways for endometrial cancer (19). “What simple, non-invasive, painless, cost-effective, and convenient tests can be used to detect cancer early?” was voted the most important research priority for Detecting Cancer Early (8). Urogenital cytology is simple, non-invasive and painless; whether it is effective for the detection of endometrial cancer is the focus of this study. We will disseminate our results through publication in peer-reviewed scientific journals, presentation at conferences and via social media, blogs and charity websites.

DISCUSSION

For peer review only - http://bmjopen.bmj.com/site/about/guidelines.xhtml

The DETECT study will establish the diagnostic test accuracy of vaginal and urine samples for endometrial cancer detection by cytology in women with PMB. Urogenital cytology could offer a simple, acceptable, easy to administer test that could be used in primary care as a triage tool for women with unexplained PMB. Cytology positive women could be referred for diagnostic work up while cytology negative women are quickly reassured without the need for unpleasant, invasive, anxiety-provoking tests, with massive cost-saving implications for the NHS.

For peer review only

Author Statement: The study was conceived and designed by EJC, who is the study guarantor. All authors contributed to the development and set up of the study. SC is responsible for the day to day running of the study, ethical/regulatory approvals and data management. ERJ, HOF, KN and CEB are responsible for patient recruitment and the collection of data and samples. NN, DS and DR are responsible for the cytological analyses. EJC provides study oversight and clinical guidance for the study. ERJ, SC and EJC drafted the manuscript. All authors contributed to, reviewed and approved the final version of the manuscript.

Funding statement: This work is funded by the Jon Moulton Charity Trust. HOF is supported by a National Institute for Health Research (NIHR) Doctoral Research Fellowship (DRF-2018-11-ST2-054). KN is supported by a Cancer Research UK Manchester Cancer Research Centre Clinical Research Fellowship (C147/A25254) and the Wellcome Trust Manchester Translational Informatics Training Scheme. CEB is supported by a Manchester University NHS Foundation Trust (MFT) Clinical Research Fellowship. EJC is supported by the NIHR Manchester Biomedical Research Centre (IS-BRC-1215-20007) and an NIHR Advanced Fellowship (NIHR300650).

Data sharing: Fully anonymised data are available from the corresponding author on reasonable request once the study is completed and main findings have been published.

Conflicts of Interest: The authors declare no conflict of interest.

For peer review only

REFERENCES

1. Cancer Research UK. Uterine cancer statistics [Accessed 30th March 2020]. Available from: https://www.cancerresearchuk.org/health-professional/cancer-statistics/statistics-by-cancer-type/uterine-cancer#heading-Zero.

2. Funston G, O'Flynn H, Ryan NAJ, Hamilton W, Crosbie EJ. Recognizing Gynecological Cancer in Primary Care: Risk Factors, Red Flags, and Referrals. Adv Ther. 2018;35(4):577-89.

3. Jones ER, O'Flynn H, Njoku K, Crosbie EJ. Detecting endometrial cancer. The Obstetrician & Gynaecologist. 2021;19 January. doi.org/10.1111/tog.12722

4. Sundar S, Balega J, Crosbie E, Drake A, Edmondson R, Fotopoulou C, et al. BGCS uterine cancer guidelines: Recommendations for practice. Eur J Obstet Gynecol Reprod Biol. 2017;213:71-97.

5. Cooper NA, Barton PM, Breijer M, Caffrey O, Opmeer BC, Timmermans A, et al. Cost-effectiveness of diagnostic strategies for the management of abnormal uterine bleeding (heavy menstrual bleeding and post-menopausal bleeding): a decision analysis. Health technology assessment (Winchester, England). 2014;18(24):1-201, v-vi.

6. Clarke MA, Long BJ, Del Mar Morillo A, Arbyn M, Bakkum-Gamez JN, Wentzensen N. Association of endometrial cancer risk with postmenopausal bleeding in women: A systematic review and meta-analysis. JAMA Internal Medicine. 2018.

7. Walker S, Hyde C, Hamilton W. Risk of uterine cancer in symptomatic women in primary care: case-control study using electronic records. The British journal of general practice : the journal of the Royal College of General Practitioners. 2013;63(614):e643-8.

8. Badrick E, Cresswell K, Ellis P, Renehan AG, Crosbie EJ. Top ten research priorities for detecting cancer early. Lancet Public Health. 2019;4(11):e551.

9. Wang Y, Li L, Douville C, Cohen JD, Yen TT, Kinde I, et al. Evaluation of liquid from the Papanicolaou test and other liquid biopsies for the detection of endometrial and ovarian cancers. Sci Transl Med. 2018;10(433).

10. Kinde I, Bettegowda C, Wang Y, Wu J, Agrawal N, Shih Ie M, et al. Evaluation of DNA from the Papanicolaou test to detect ovarian and endometrial cancers. Sci Transl Med. 2013;5(167):167ra4.

11. Bakkum-Gamez JN, Wentzensen N, Maurer MJ, Hawthorne KM, Voss JS, Kroneman TN, et al. Detection of endometrial cancer via molecular analysis of DNA collected with vaginal tampons. Gynecologic oncology. 2015;137(1):14-22.

For peer review only

12. Frias-Gomez J, Benavente Y, Ponce J, Brunet J, Ibáñez R, Peremiquel-Trillas P, et al. Sensitivity of cervico-vaginal cytology in endometrial carcinoma: A systematic review and meta-analysis. Cancer Cytopathol. 2020.

13. Njoku K, Chiasserini D, Jones ER, Barr CE, O'Flynn H, Whetton AD, Crosbie EJ. Urinary Biomarkers and Their Potential for the Non-Invasive Detection of Endometrial Cancer. Front Oncol. 2020 Nov 3;10:559016.

14. O'Flynn H, Ryan NAJ, Narine N, Shelton D, Rana D, Crosbie EJ. Diagnostic accuracy of cytology for the detection of endometrial cancer in urine and vaginal samples. Nature Communications. 2021. Feb 11;12(1):952.

15. Bossuyt PM, Reitsma JB, Bruns DE, Gatsonis CA, Glasziou PP, Irwig L, et al. STARD 2015: an updated list of essential items for reporting diagnostic accuracy studies. Bmj. 2015;351:h5527.

16. Rovers Medical Devices. Delphi Screener [30th March 2020]. Available from: https://www.roversmedicaldevices.com/cell-sampling-devices/delphi-screener/.

17. FIGO Committee on Gynecologic Oncology. FIGO staging for carcinoma of the vulva, cervix, and corpus uteri. International Journal of Gynecology & Obstetrics. 2014;125(2):97-8.

18. Kurman RJ, Carcangiu ML, Herrington CS, Simon C. World Health Organisation Classification of Tumours of Female Reproductive Organs. International Agency for Research on Cancer 2014;4th Revised ed.

19. Wan YL, Beverley-Stevenson R, Carlisle D, Clarke S, Edmondson RJ, Glover S, et al. Working together to shape the endometrial cancer research agenda: The top ten unanswered research questions. Gynecologic oncology. 2016;143(2):287-93.

For peer review only

Figure legends

Figure 1: Study schema illustrating the flow of participants through the study, interventions and evaluations

Figure 2: Diagnostic pathway for women referred to secondary care for the urgent investigation of unexplained PMB

For peer review only

For peer review only - http://bmjopen.bmj.com/site/about/guidelines.xhtml

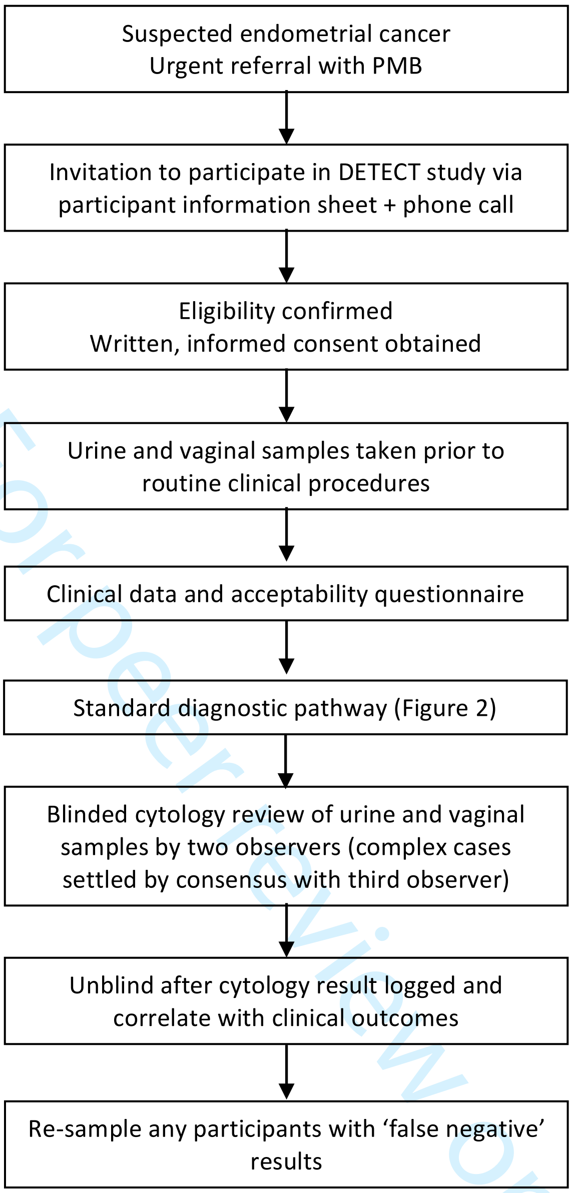

For peer review only

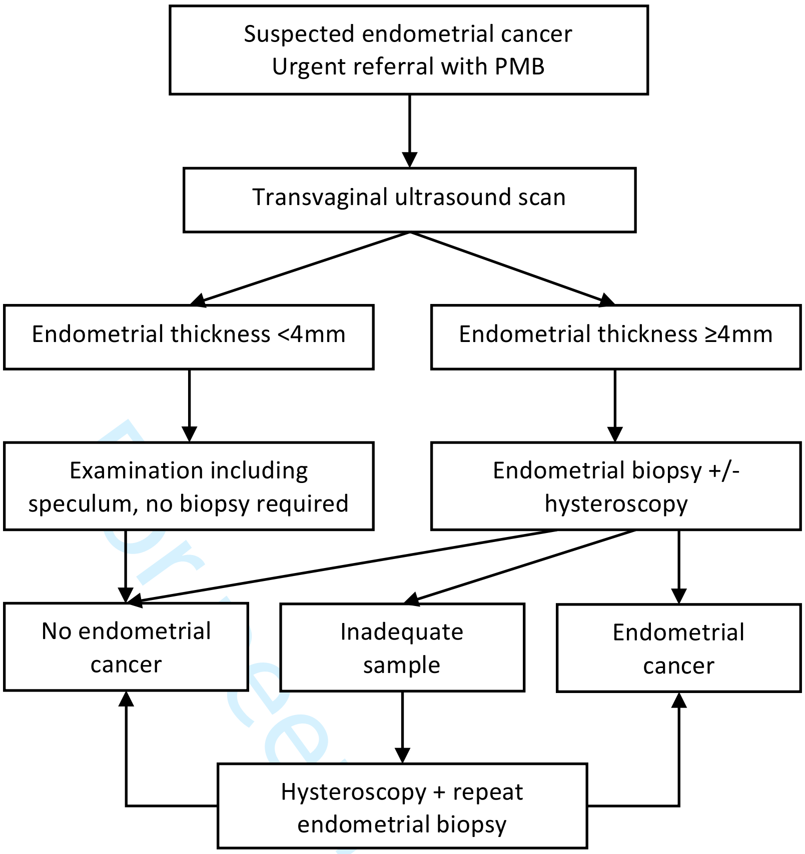

For peer review only

For peer review only

For peer review only - http://bmjopen.bmj.com/site/about/guidelines.xhtml

Appendix 1. Acceptability questionnaire

How would you describe your education level?

Primary School (Up to age 11) ☐

Secondary school (Up to age 16) ☐

Higher Education or above (A Levels/ University) ☐

	Yes	No
**Have you used any vaginal creams/ pessaries before?**	☐	☐
**Have you ever used tampons?**	☐	☐
**Have you given a urine sample before?**	☐	☐
**Have you ever used a home pregnancy test?**	☐	☐

Have you been sexually active?

Within the last month ☐

Within the last year ☐

More than a year/ not sexually active ☐

Would rather not say ☐

How would you describe the amount of discomfort experienced during the vaginal washing?

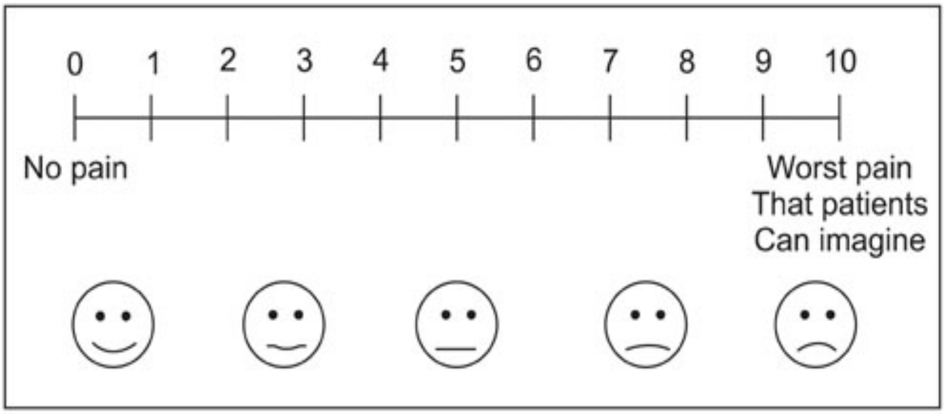

For peer review only

How would you describe the amount of discomfort experienced during pelvic examination?

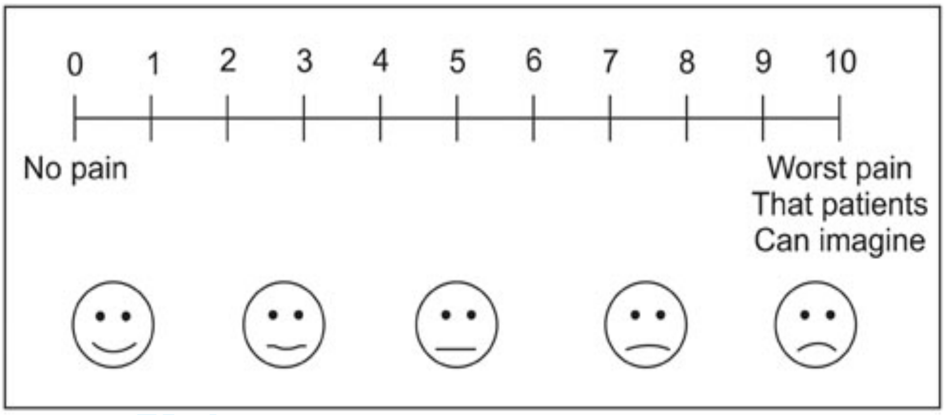

How would you describe the amount of discomfort experienced during ultrasound scan?

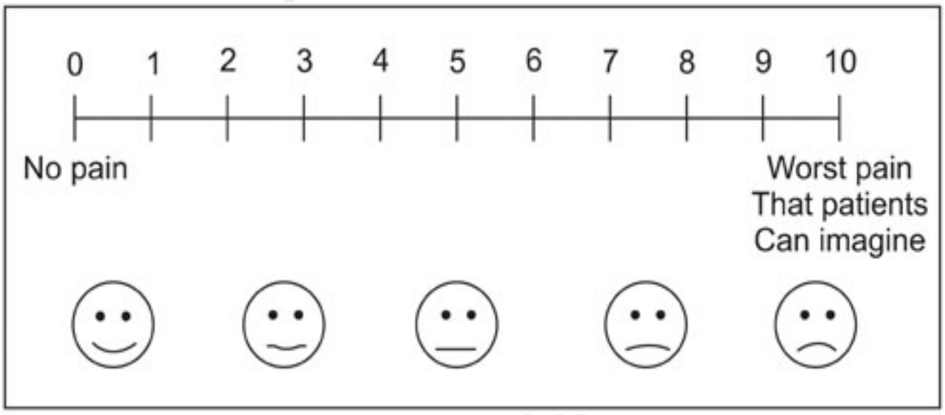

How would you describe the amount of discomfort experienced during hysteroscopy?

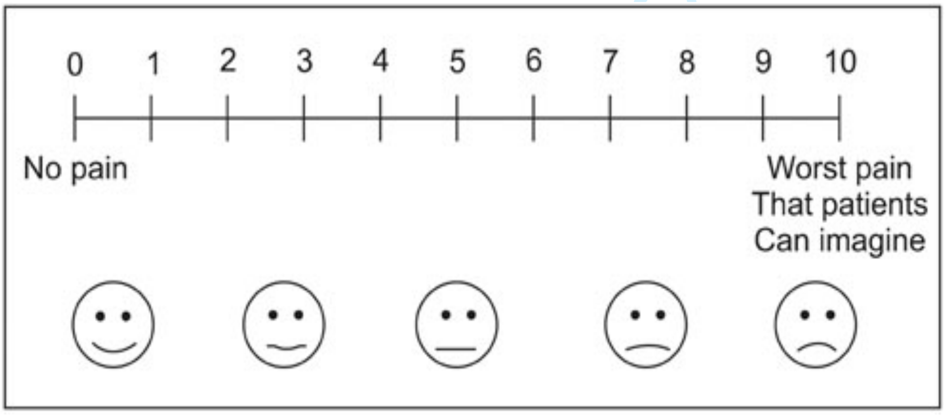

For peer review only - http://bmjopen.bmj.com/site/about/guidelines.xhtml

How would you describe the amount of discomfort experienced during endometrial biopsy sampling?

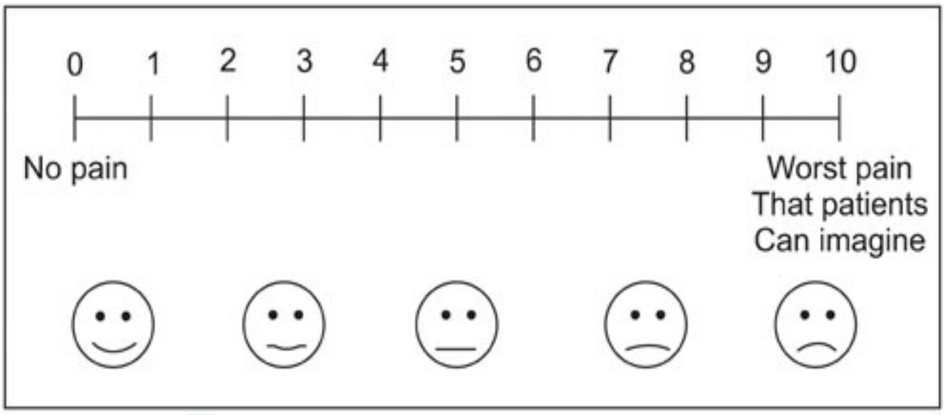

If needed how likely are you to have the following investigations again?

	**Definitely not**	**Probably not**	**Not sure**	**Probably**	**Definitely**
Ultrasound scan	☐	☐	☐	☐	☐
Vaginal washing	☐	☐	☐	☐	☐
Urine sample	☐	☐	☐	☐	☐
Pelvic examination	☐	☐	☐	☐	☐
Hysteroscopy	☐	☐	☐	☐	☐
Endometrial biopsy	☐	☐	☐	☐	☐

For peer review only
